# Dealkylation of Macromolecules by Eukaryotic α-Ketoglutarate-Dependent Dioxygenases from the AlkB-like Family

**DOI:** 10.3390/cimb46090622

**Published:** 2024-09-20

**Authors:** Anastasiia T. Davletgildeeva, Nikita A. Kuznetsov

**Affiliations:** 1 Institute of Chemical Biology and Fundamental Medicine, Siberian Branch of Russian Academy of Sciences, Novosibirsk 630090, Russia; nikita.kuznetsov@niboch.nsc.ru; 2 Department of Natural Sciences, Novosibirsk State University, Novosibirsk 630090, Russia

**Keywords:** macromolecule alkylation, dealkylation, dioxygenase, catalytic mechanism, single-nucleotide polymorphism, enzyme disfunction

## Abstract

Alkylating modifications induced by either exogenous chemical agents or endogenous metabolites are some of the main types of damage to DNA, RNA, and proteins in the cell. Although research in recent decades has been almost entirely devoted to the repair of alkyl and in particular methyl DNA damage, more and more data lately suggest that the methylation of RNA bases plays an equally important role in normal functioning and in the development of diseases. Among the most prominent participants in the repair of methylation-induced DNA and RNA damage are human homologs of *Escherichia coli* AlkB, nonheme Fe(II)/α-ketoglutarate-dependent dioxygenases ABH1–8, and FTO. Moreover, some of these enzymes have been found to act on several protein targets. In this review, we present up-to-date data on specific features of protein structure, substrate specificity, known roles in the organism, and consequences of disfunction of each of the nine human homologs of AlkB. Special attention is given to reports about the effects of natural single-nucleotide polymorphisms on the activity of these enzymes and to potential consequences for carriers of such natural variants.

## 1. Alkylation of DNA, RNA, and Proteins and Potential Mechanisms Underlying the Repair of Such Modifications

Along with oxidative DNA damage, spontaneous or enzymatic depurination/depyrimidination of nucleotides, erroneous incorporation of dNTPs into DNA, and alkylating DNA lesions induced by exogenous chemical agents or endogenous metabolites represent major types of DNA damage in the cell [1,2,3,4]. DNA alkylation is the result of the addition of a new alkyl group at a noncanonical position of DNA from a donor of the alkyl group by monomolecular or bimolecular nucleophilic substitution reactions. Oxygen atoms of the phosphodiester backbone or O/N atoms of DNA nucleobases can all be possible recipients of the alkyl group. Methylation (transfer of the methyl group) is the most common type of alkyl modification [5].

Repair of alkylation-induced lesions is crucial for all cell types because such damage is cytotoxic and potentially mutagenic. Alkylating chemotherapy is a common treatment of many tumors, implying the importance of alkylation damage repair pathways in tumor cells [6]. On the other hand, methylation is one of the most widespread types of epigenetic modifications in living organisms [7]. DNA methylation is a covalent modification that regulates the expression of genes via a number of mechanisms, including the binding of transcription factors [8] and recruitment of methylated DNA binding proteins and chromatin-modifying proteins, all of which leads to changes in a chromatin state [9]. Methylation of DNA at the C5 position of cytosine (m^5^C) in the context of CpG dinucleotides underlies modulation of many biological processes, including gene expression, suppression of retrovirus expression, X-chromosome inactivation, and several other vital functions in mammalian cells [10]. Moreover, it has been shown that C5 methylation of cytosine in CpG dinucleotides leads to substantial alterations of local DNA shape [11,12,13]. Aberrations in DNA methylation patterns cause diverse disorders ranging from cancers and immunological diseases to various neurological and mental disorders [14,15,16,17,18,19]. Aside from its undoubted and substantial role as an epigenetic mark, m^5^C is also one of the major sources of substitutions C>T and G>A in human DNA [20].

Similar to DNA, RNA can also be methylated by different endogenous and exogenous agents in the cell. The methylation of RNA bases has been shown to have a negative impact on the process of mRNA decoding [21,22,23,24]. In this context, considering the complex nature of the decoding process (during which three nucleotides are read simultaneously), methylation-induced modifications in mRNA may have complicated effects (on transfer RNA [tRNA] selection) that cannot be predicted solely from their influence on the formation of complementary codon–anticodon interactions. Furthermore, chemical damage to noncoding RNAs—which constitute approximately 95% of all cellular RNA and include ribosomal RNA (rRNA), tRNA, small nuclear RNAs, and small nucleolar RNAs—may affect their function too [25,26,27]. Indeed, it is not yet fully understood how the cell copes with RNA damage. Scientific data indicate that some quality control processes exist in the cell that are capable of recognizing and degrading or repairing damaged RNA [28,29].

In fact, processes underlying the restoration of the original structure in biopolymers remain the best studied for DNA and have been investigated to a much lesser extent for RNA. Proteins, just as RNAs, are temporary molecules, and when damaged, they are usually degraded by proteasomes or autophagy [30,31,32]. Nonetheless, it should be noted that there are known examples of the restoration of modified proteins. For instance, it has been established that methionine oxidation, giving rise to methionine sulfoxide [33], can be reversed by methionine sulfoxide reductase [34]. Protein methylation is a common post-translational modification, and either the N terminus or C terminus of a protein or side-chain nitrogen atoms of lysine and arginine residues can be methylated [35,36,37,38,39]. A special case of protein methylation is site-specific methylation or demethylation of histones, which is catalyzed by the corresponding methyltransferases and demethylases [40]. Therefore, it can be hypothesized that the cell may have pathways for the repair of alkylation-induced modifications that are common among amino acid residues (aa).

In this review, using eukaryotic representatives of the family of AlkB-like nonheme Fe(II)/α-ketoglutarate-dependent dioxygenases, which are responsible for the dealkylation of macromolecules in the cell, we examine the functional characteristics and biological roles of these enzymes in dealkylation processes. For each AlkB human homolog, specific features of protein structure, substrate specificity, known role in the organism, and known consequences of disruption of these enzymes’ functioning are discussed. Special attention is given to reports about effects of natural single-nucleotide polymorphisms on the activity of these enzymes and to potential consequences for carriers of such natural variants.

## 2. S-Adenosylmethionine (SAM)-Dependent Endogenous Methylation

**Figure 1:**
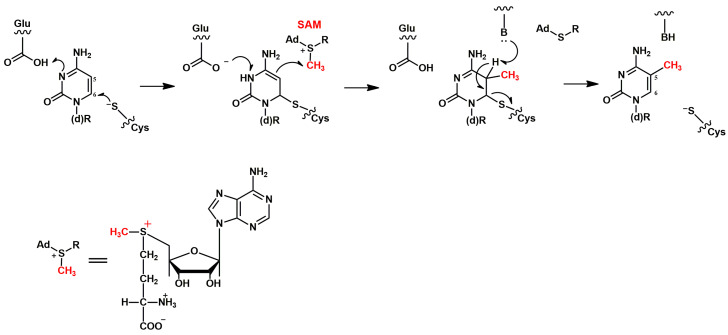
The mechanism of enzymatic methylation of a DNA base (cytosine) by SAM, as catalyzed by DNA methyltransferases (DNMTs). A conserved cysteine residue of a DNMT mediates a nucleophilic attack on the C6 atom of the cytosine ring, and this event initiates the reaction. This attack is also facilitated by a conserved glutamic acid residue. After this attack, transfer of a methyl group from SAM to atom C5 of the cytosine ring takes place. Subsequent deprotonation of C5 resulting in m^5^C formation is thought to be mediated by a basic group (presented as “B:”) provided by the enzyme. The mechanism is based on data from [42].

In addition to SAM, no other obvious candidates for the role of intracellular alkylating compounds have been identified to date (Figure 1). SAM serves as a donor in most of enzymatic methylation reactions in vivo by transferring a methyl group to diverse acceptors. Meanwhile, the strong ability of SAM to transfer the methyl group inevitably causes spontaneous methylation of cellular nucleic acids and proteins [4,41].

**Figure 2:**
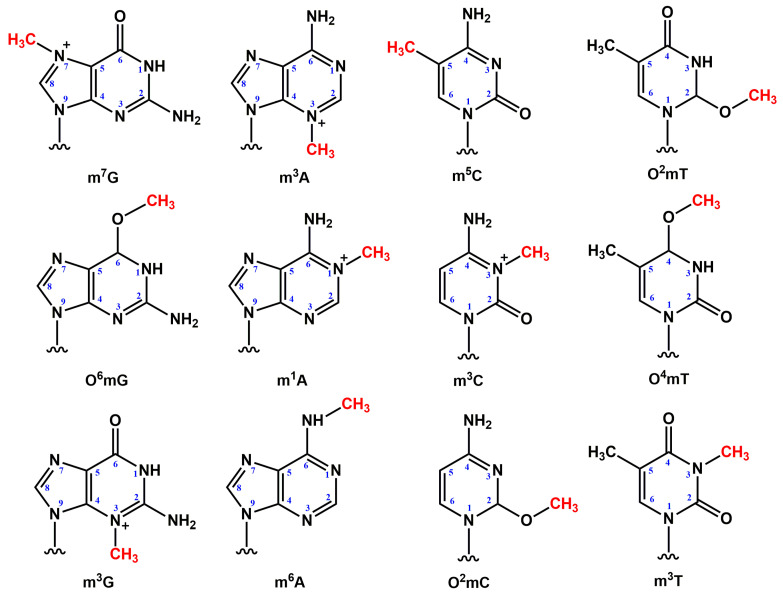
Structures of the methylated adducts of nucleic acid bases. Presented methylated guanine derivatives are *N*^7^-methylguanine (m^7^G), *O*^6^-methylguanine (O^6^mG), and *N*^3^-methylguanine (m^3^G); adenine derivatives are *N*^3^-methyladenine (m^3^A), *N*^1^-methyladenine (m^1^A), and *N*^6^-methyladenine (m^6^A); cytosine derivatives are 5-methylcytosine (m^5^C), *N*^3^-methylcytosine (m^3^C), and *O*^2^-methylcytosine (O^2^mC); thymine derivatives are *O*^2^-methylthymine (O^2^mT), *O*^4^-methylthymine (O^4^mT), and *N*^3^-methylthymine (m^3^T). Methylation modifications are indicated in red.

During a reaction of double-stranded DNA (dsDNA) with SAM, the main products are *N*^7^-methylguanine (m^7^G) and *N*^3^-methyladenine (m^3^A, Figure 2). In this context, the emergence of the latter in DNA, in contrast to m^7^G, exerts a toxic effect. Instead of participating in the formation of normal Watson–Crick bonds with a complementary base, the methyl group of m^3^A ends up in the minor groove of DNA, thereby effectively blocking the binding of most RNA and DNA polymerases. Usually, the minor groove is free of methyl groups because methyl groups of thymine and m^5^C are positioned in the major groove. Thus, the emergence of m^3^A in DNA has a strong cytotoxic impact but a weak mutagenic effect [4].

Although the role of SAM in the methylation of single-stranded DNA (ssDNA) has not been researched in detail in early studies, some other alkylating agents have been shown to induce ssDNA lesions such as *N*^1^-methyladenine (m^1^A) and *N*^3^-methylcytosine (m^3^C, Figure 2) [43,44]. These modified bases are also unable to form classic bonds with a complementary base and thus block DNA replication.

Given that research attention has been focused on m^5^C as the best-studied and best-understood epigenetic mark in DNA, another potential epigenetic mark, *N*^6^-methyladenine (m^6^A, Figure 2), has been mostly ignored until recently. The main reason is that in the past, m^6^A has been detected only in prokaryotic genomes [45]. In the last decade, however, the presence of m^6^A has been registered in the DNA of numerous eukaryotic species [45], including popular model organisms such as *Caenorhabditis elegans* [46,47], *Arabidopsis thaliana* [48], and *Drosophila* [49,50] as well as in humans [51,52,53]. Of note, recent single-molecule real-time sequencing proved that modification m^6^A is widespread in the human genome [53], including the mitochondrial genome [51]. Additionally, m^6^A levels fluctuate dramatically during early embryogenesis [54].

More and more research articles indicate that m^6^A plays an important role in eukaryotes. Nevertheless, the enzymes participating in the installation and removal of this mark remain poorly investigated [45]. The methylation of adenosine in DNA at the sixth position may also involve SAM. The m^6^A methyltransferases discovered in eukaryotes mainly belong to the family of MT-70 methyltransferases, such as methyltransferase like 4 (METTL4) in most mammals and DAMT-1 in *C. elegans* [45]. On the other hand, the only currently known mammalian m^6^A DNA demethylase is ABH1, which is a homolog of bacterial AlkB [55].

At the same time, there is no consensus in the scientific community regarding the role of m^6^A in the mammalian genome. For instance, Douvlataniotis et al. have reported that the totality of their experimental evidence indicates that the data published to date are insufficient to confirm the presence of m^6^A in the mammalian genome [56]. Thus, further effort from researchers will be needed to determine the true position of the m^6^A mark in epigenetics. Investigation into enzymatic systems responsible for the m^6^A emergence in DNA and for m^6^A removal, as well as the pathways controlling the competitive impact of these systems may shed a light on the role of this modification.

## 3. Methylation by Exogenous Agents

The interaction of methylating agents with DNA leads to the formation of adducts of bases, which are methylated either on nitrogen atoms of the base’s ring or on exocyclic atoms [57]. The most harmful alkylation-induced lesions in dsDNA are *O*^6^-methylguanine (O^6^mG) and m^3^A (Figure 2). O^6^mG, whose presence in DNA can directly cause mutations, is rapidly demethylated by *O*^6^-methylguanine DNA methyltransferase (MGMT), which is an enzyme conserved from bacteria to humans [58,59]. O^6^mG is a major methylation product during treatment of DNA with methylating agents or chemotherapeutic drugs such as temozolomide [60]. In addition, O^6^mG, which is highly mutagenic during DNA replication, has been found to affect the rate and accuracy of mRNA decoding during translation. The effect of O^6^mG on translation depends on its position within a codon [22].

Other methylated lesions, including m^3^C, can also arise during treatment of DNA with methylating agents or chemotherapeutic drugs, but their proportion is much smaller, and they are much less studied. Right now, there are no known enzymatic pathways for the formation of m^3^C in DNA, and therefore it is believed that this lesion can be formed only chemically, with SAM or methyl methanesulfonate (MMS) being possible sources of the methyl group [61]. m^3^C predominantly forms in ssDNA and RNA; consequently, cells with higher transcription rates are more susceptible to this lesion because their DNA is in a single-stranded state more often [61]. In their study, Furrer and van Loon examined the ability of six DNA polymerases belonging to the B, X, and Y families (namely, Pol δ, β, λ, κ, ι, and η) to bypass an m^3^C lesion in DNA [62]. Among all the tested enzymes, Pol η showed the highest efficiency of bypassing this lesion. Moreover, on substrates with a hanging 5’ end or substrates with a long gap, Pol η inserted opposite m^3^C any of the four nucleotides with different preferences. By contrast, on substrates with a single-nucleotide gap, only A or G were inserted opposite m^3^C [62].

m^3^A in DNA blocks replication and is hence a cytotoxic lesion, but sometimes the emergence of this lesion in DNA can lead to mutations if DNA polymerase manages to go through the lesion. To prevent such adverse consequences, m^3^A is removed from cell DNA by 3-alkyladenine-DNA-glycosylase (AAG) via the base excision repair pathway [63]. m^7^G, one of major products of SAM’s action on DNA, is a rather common and relatively harmless modification of a DNA base. m^7^G gradually disappears from DNA owing to spontaneous hydrolysis as well as due to the activity of AAG [63]. Some less common methylation-induced DNA lesions, including *N*^3^-methylguanine (m^3^G), *O*^2^-methylthymine (O^2^mT), and *O*^2^-methylcytosine (O^2^mC), are removed from DNA by *Escherichia coli* 3-methyladenine-DNA-glycosylase AlkA, whereas *O*^4^-methylthymine (O^4^mT, Figure 2) is removed by the MGMT enzyme in *E. coli* and humans [64].

m^1^A and m^3^C, being major methylation-induced lesions forming in ssDNA [44,57], are thought to arise in vivo during such processes as replication and transcription, thereby blocking the movement of DNA and RNA polymerases. These lesions are stable and do not appear to be removed by DNA glycosylases [65]. *N*^3^-methylthymine (m^3^T, Figure 2) is also a noncoding lesion and blocks subsequent DNA synthesis. In addition, this lesion is chemically extremely stable [57]. The removal of such damage is performed by enzymes belonging to the superfamily of Fe(II)/α-ketoglutarate (αKG)-dependent dioxygenases. This superfamily of enzymes uses oxidative degradation of αKG to hydroxylate their target substrates [65,66,67,68,69,70].

## 4. Eukaryotic Dioxygenases of the AlkB Family as Universal Dealkylating Enzymes

The AlkB enzyme, first discovered in *E. coli*, can counteract alkylating damage in DNA and restore its native structure. With the discovery of its homologs in eukaryotes, especially in humans, its role as a demethylase is becoming increasingly visible and important.

Because the list of possible substrates of AlkB homologs continues to expand, research in this field has also been rapidly developing in recent decades, including (i) an investigation into polymorphisms in genes of these enzymes and into their involvement in signaling pathways and (ii) an analysis of functionally significant regions of these proteins [71,72,73,74,75,76].

**Figure 3:**
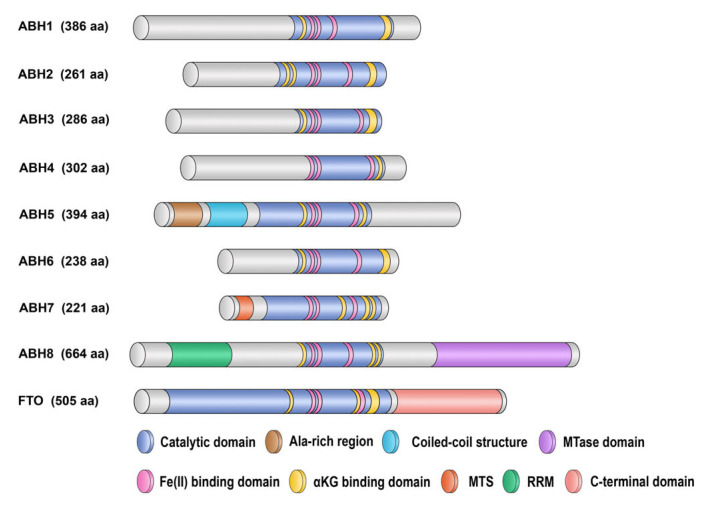
Distribution of key functional elements in ABH enzymes. The catalytic domain (steel blue) includes Fe(II)- and αKG-binding sites (pink and yellow sectors, respectively), and these sites are completely conserved among the nine AlkB human homologs. In ABH5 structure, an amino-terminal alanine-rich region and a potential coiled-coil structure are distinguished, which could be important for its localization [76]. ABH7 also has a specific nonconserved region: a mitochondrial targeting signal (MTS, orange) [84]. ABH8 has an additional RNA recognition motif (RRM, green) and a C-terminal methyltransferase (MT) domain (purple section) [85]. FTO consists of an N-terminal domain, including the catalytic domain, and of a C-terminal domain (interacting with the first one) [86,87]. This image was based on data from [74,76,88].

It has been established that the *E. coli* enzyme AlkB removes alkyl damage from DNA bases by oxidizing them [4,69]. According to the catalytic mechanism of action, all representatives of the family of AlkB-like enzymes belong to the superfamily of Fe(II)/αKG-dependent dioxygenases [66,67,69]. Homologs of AlkB are widespread among eukaryotes and take part in the demethylation of a wide range of substrates, including DNA, RNA, and proteins [77,78]. To date, nine mammalian homologs of AlkB are known, namely ABH1–8 and FTO (fat mass and obesity-associated protein, also known as ABH9; Figure 3) [79,80,81,82]. Of the nine eukaryotic enzymes, ABH1 is the most closely related to AlkB in humans [81]. Nevertheless, each of these enzymes contains a functional catalytic domain characteristic of dioxygenases. On the other hand, these enzymes are localized to different regions of the cell, catalyze the dealkylation of different substrates, and therefore have different biological roles. Some of the nine homologs, in particular ABH5 and FTO, have been extensively studied as RNA demethylases [15,72,83]. For some of these enzymes, however, such as ABH4, ABH6, and ABH7, there are very few data on their substrate specificity and functions in the organism.

### 4.1. ABH1

ABH1 has apurinic/apyrimidinic (AP) lyase and nucleic acid demethylase activities, and the AP-lyase activity of ABH1 is independent of Fe(II) and αKG [89]. Despite its high homology to *E. coli* AlkB, ABH1 is longer by 173 aa (71 at the N terminus, 42 at the C terminus, and one residue somewhere in the middle). The full-length sequence of ABH1 is highly conserved among humans, mice, and chickens (70–83% identity), suggesting that the extensions have functional significance.

Notably, Abh1 (*Schizosaccharomyces pombe* dioxygenase), the most homologous to human ABH1 (among all human homologs of AlkB; 42% homology), despite having weak AP-lyase activity in vitro, does not exert any demethylase action [90].

Data on substrate specificity of ABH1 in the literature remain sparse. ABH1 is reported to have weak activity toward m^3^C in RNA and ssDNA [91,92]. In addition, one study suggests that ABH1 can oxidize m^5^C in RNA [93]. Not so long ago, it was found that ABH1 can also demethylate m^1^A in tRNA, thus participating in translation [94,95].

ABH1 is also the only currently known demethylase of m^6^A in DNA; this modification was recently recognized by many scientists as another important epigenetic mark [55]. Lately, it has been demonstrated that ABH1 removes m^6^A from DNA both in a cell-free system [96] and in several cellular models [55]. Evidently, ABH1 removes m^6^A in unpaired regions of the mammalian genome by preferring loops or bubbles in DNA [55].

*Abh1* knockout mice (*Abh1*^(^^−^^/−)^ mice) have been created, with one study revealing the development of a specific phenotype associated with deficiencies in the differentiation of placental trophoblast lineages [97]. In a later study, the results are suggestive of an important role of Abh1 in spermatogenesis and embryonic development because *Abh1*^(−/−)^ mice exhibit incomplete penetrance phenotypes, including unilateral eye malformations, neural-tube defects, and craniofacial and skeletal abnormalities. Nonetheless, ~10% of *Abh1*^(−/−)^ mice appear to be relatively normal, while the most affected mice die early in embryogenesis [98].

**Figure 4:**
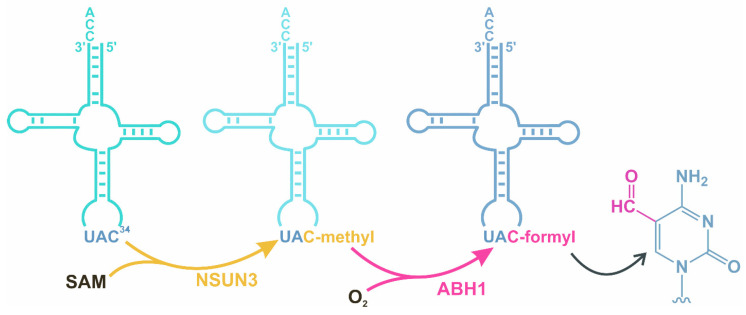
The anticodon of mitochondrial tRNA^Met^ is modified by two sequential enzymatic reactions. At the first step, cytosine at position 34 (at the wobble position of the anticodon) is methylated by NSUN3 in a reaction involving SAM. Then, ABH1 converts m^5^C to formylcytosine, thereby extending the spectrum of recognized codons to AUG, AUA, and AUU. This image was based on data from [99].

ABH1 is reported to participate in the modification of mitochondrial tRNAs. In the biosynthetic pathway that introduces modifications into the wobble position in mitochondrial tRNA^Met^, RNA methyltransferase NSUN3 methylates C34 of mitochondrial tRNA^Met^ thus generating m^5^C, which can then be oxidized by ABH1 (Figure 4) [93]. The formylcytosine produced by this reaction is required at this position to mediate the binding of mitochondrial tRNA^Met^ to AUG, AUA, and AUU [99].

In mouse embryonic stem cells, m^6^A, which is regulated by ABH1, is abundant in transposon LINE-1 and inhibits its transcription [52]. Furthermore, in a recent study, Li et al. demonstrated that ABH1 takes part in the regulation of sensory axon regeneration, thus shedding light on the important physiological function of ABH1 and m^6^A in DNA [100].

A growing number of articles suggest that the expression level of ABH1 can be employed as a marker for a more accurate and rapid diagnosis of cancers [91,101,102]. Wang et al. [103] have shown that ABH1 serves as an oncogenic protein by suppressing the formation of m^6^A, thus leading to proliferative and metastatic properties in vitro and in vivo. An *Abh1* knockout in mice aggravates the progression of gastric cancer in a chemically induced tumorigenesis model. In that study, it was determined that ABH1-mediated demethylation of m^6^A in gastric cancer cells selectively affects transcriptional activity of NRF1, thereby attenuating the launch of AMP-activated protein kinase (AMPK) signaling and promoting the Warburg phenotype in gastric cancer cells [103].

It has been revealed that an overexpression of ABH1 correlates with poor outcomes in gastric adenocarcinoma by affecting both the tumor microenvironment and macrophage infiltration [104]. Furthermore, according to some data, ABH1 participates in the tumorigenesis of gastric cancer and glioblastoma [105,106].

In a recent work, Zhang et al. [107] carried out SNP genotyping of ABH1 in 402 patients with neuroblastoma and in 473 healthy controls by TaqMan analysis. Based on the results, it was theorized that substitution rs2267755 C>T in the 3′-untranslated region (UTR) of the *ABH1* gene is a genetic variant that reduces the risk of neuroblastoma [107].

With a growing body of evidence that the regulation of m^6^A levels in DNA is important in terms of carcinogenesis and with the only enzyme known to participate being ABH1, it is becoming clear that this field of research must attract the attention of scientists in the near feature. For example, in the last few years, Xiong et al. developed the first potent and selective small-molecule inhibitor of ABH1, implying a strong interest of the scientific community in this enzyme as a modulator of potentially important epigenetic mark m^6^A [55]. At the same time, little is known about the role of the ABH1 activity in physiology. There are almost no data about the SNPs of ABH1 and the physiological consequences of the interruption of the activity of this enzyme. There is a possibility that the absence of known polymorphisms is due to the extreme importance of ABH1 for development, although experiments with mice on this subject do not show 100% lethality in *Abh1* knockout mice [98]. Considering the increasing interest in ABH1 in light of its connection to m^6^A, there is hope that the amount of data on this enzyme’s polymorphisms, changes in expression levels, and roles in the human organism will grow.

### 4.2. ABH2 and ABH3

**Figure 5:**
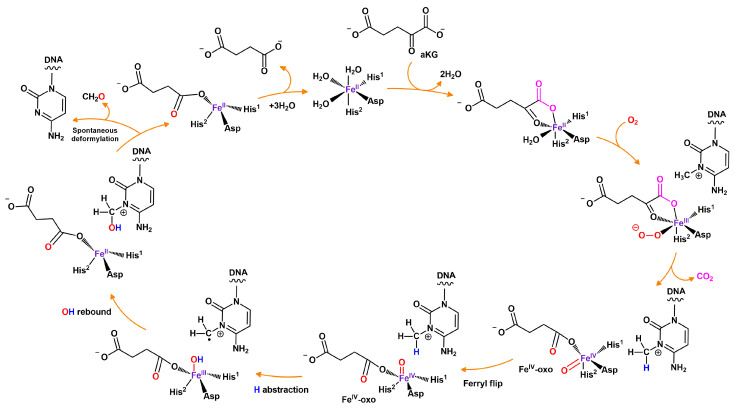
A proposed mechanism of catalytic oxidation of a methylated substrate by AlkB family enzymes, presenting m^3^C oxidation as an example. The mechanism used by AlkB family dioxygenases to oxidize the methyl group of DNA bases consists of two main stages, namely, dioxygen activation and substrate oxidation itself. Before binding an oxygen molecule, iron is coordinated by two histidine residues (His1 and His2) and aspartate, whereas αKG is coordinated in the enzyme’s active center. Free Fe(II) coordination sites are usually occupied by water molecules. Binding of the substrate in the active center initiates the reaction. During the first stage, αKG is oxidized to succinate and CO_2_. In this way, a reactive Fe(IV) = O intermediate is formed. In the second stage, the methyl group is hydroxylated by this activated intermediate. Restoration of the original structure of the substrate (cytosine here) proceeds through spontaneous deformylation. This mechanism was based on data from [114,115].

Enzymes ABH2 and ABH3 are in the same clade of the phylogenetic tree, and therefore discussions of one often include the other. As all other Fe(II)/αKG-dependent dioxygenases [66,67,69], ABH2 and ABH3 use nonheme Fe(II) as a cofactor and αKG to oxidize a noncanonical alkyl group of a substrate base (Figure 5). ABH2 prefers dsDNA, whereas ABH3 prefers ssDNA and mRNA [79,108,109,110]. Unlike many other DNA repair enzymes, ABH2 has quite a broad spectrum of substrate specificity encompassing m^1^A, m^3^C, and some exocyclic adducts in genomic DNA [111]. ABH3 has activity toward m^1^A and m^3^C too [79] and weak activity toward 3,*N*^4^-ethenocytosine (εC) in the context of ssDNA [112]. A recent paper also shows an ability of ABH2 and ABH3 to remove epigenetic mark m^5^C from ssDNA and dsDNA in vitro [113].

Aside from methylation of adenosine at position 1 or 6, double adenosine modifications such as *N*^6^,*N*^6^-dimethyladenosine (m^6,6^A) and 1,*N*^6^-dimethyladenosine (m^1,6^A) have been detected in mammalian RNA [116]. The same authors also demonstrated that ABH3, but not ABH1, can remove m^1,6^A from tRNA.

Ringvoll et al. have reported that mice lacking *Abh2*, *Abh3*, or both functional genes are viable and have no altered phenotypes [110]. Nevertheless, in the absence of any exposure to exogenous methylating agents, mice devoid of ABH2, but not mice with ABH3 deficiency, accumulate appreciable amounts of m^1^A in the genome, indicating the presence of a biologically significant endogenous methylating agent. Furthermore, embryonic fibroblasts from ABH2-deficient mice fail to remove MMS-induced m^1^A from their genomic DNA and undergo enhanced toxic effects after exposure to MMS. Thus, the repair of m^1^A and m^3^C in vitro (in nuclear extracts) in dsDNA depends primarily, if not exclusively, on ABH2 [110].

Li et al. [117] have highlighted an important role of ABH2 in the maintenance of the integrity and transcription of ribosomal DNA (rDNA). In the nucleolus of mammalian cells, ABH2 is present at high concentrations and this protein interacts with resident nucleolar proteins nucleolin (NCL), nucleophosmin 1 (NPM1), and upstream binding factor (UBF) and with DNA repair proteins Ku70 and/or Ku80 and binds to rDNA genes. ABH2 regulates rDNA transcription in an enzymatic activity-dependent manner, whereas an ABH2 knockdown results in a significant increase in DNA damage, especially in rDNA genes. This evidence points to an important function of ABH2—for the transcription and integrity of rDNA—in the repair of DNA lesions caused by endogenous alkylation [117].

ABH3 is currently regarded as the main enzyme responsible for the removal of m^1^A from RNA [118]. Lately, more and more data have been coming out on the role of emergence of modification m^1^A in nucleic acids within carcinogenesis. ATP5D, one of the most important subunits of adenosine-5-triphosphate synthase, partakes in tumor cell glycolysis, which is regulated by m^1^A demethylase ABH3. m^1^A-modified A71 in exon 1 of *ATP5D* negatively regulates translation elongation of *ATP5D* mRNA by increasing binding to the YTHDF1–eRF1 complex, thus facilitating the release of this mRNA from the ribosomal complex. m^1^A also modulates the stability of *E2F1* mRNA, which directly binds to the *ATP5D* promoter, thereby initiating its transcription. Additionally, ABH3 is being investigated as a participant in the regulation of these processes owing to its m^1^A demethylase activity toward RNA [118].

On the other hand, demethylation of tRNA at m^1^A makes it more sensitive to cleavage by angiogenin (ANG), after which tRNA-derived small RNAs (tDRs) form around anticodon regions. tDRs are conserved among different species, can enhance ribosome assembly, and prevent the apoptosis triggered by cytochrome C. Chen et al. have revealed that ABH3 can promote proliferation, migration, and invasiveness of tumor cells owing to its ability to oxidize m^1^A and m^3^C in tRNA [119].

A polymorphic variant of ABH2 containing the I141V substitution has been detected in a patient with glioma. Amino acid residue 141 of the ABH2 enzyme is located in a region conserved in homologs between *E. coli*, mice, and humans [120]. In addition, Fu et al. [121] have investigated two polymorphic variants of ABH2 that contain substitution A9V or Q10K in the PCNA-binding region and are associated with cancers, according to the Catalog of Somatic Mutations in Tumors (COSMIC) database. It was demonstrated in that work that the two substitutions, while insignificantly affecting the catalytic activity in relation to methylated dsDNA substrates, either weaken (in the case of A9V) or significantly enhance the affinity of ABH2 for PCNA (in the case of the polymorphic variant Q10K) [121].

ABH2 downregulation has been found to inhibit epithelial–mesenchymal transition [122], and a similar regulatory effect has been detected in colorectal cancer cell lines, where an ABH2 knockdown inhibits the proliferation and invasive capacity of colorectal cancer cells through upregulation of E-cadherin and decreased expression of N-cadherin [123]. Downregulation of ABH2 also increases chemotherapy sensitivity of non-small cell lung cancer cell lines [124]. Moreover, ABH2 upregulation in human glioblastoma cell lines strengthens chemotherapy resistance [125].

In general, it can be said that at the moment, there are very few data on polymorphisms or disruption of regulation for ABH2. Nonetheless, considering the exclusive role of this enzyme in the protection of rDNA from alkylating damage, much more effort should be made to study the participation of ABH2 in the development of various diseases, including in carcinogenesis. The attenuating effects of ABH2 downregulation on the proliferation of cancer cells and on chemotherapy resistance also indicate that this enzyme could be a promising target for the design of inhibitors for anticancer therapy.

ABH3 seems to play an important part in the survival of lung adenocarcinoma cells and prostate adenocarcinoma cells [126,127]. Lately, some authors have also been investigating and optimizing benzimidazole-based inhibitors of ABH3 dioxygenase. These studies have yielded several derivatives that inhibit the proliferation of prostate cancer DU145 cells in culture [128,129].

Wang et al. have shown that ABH3 is overexpressed in hepatocellular carcinoma compared to adjacent nontumorous tissue samples [130]. Moreover, in that paper, ABH3 expression was found to strongly correlate with tumor differentiation status and the tumor–node–metastasis stage. An ABH3 knockdown inhibited the proliferation of hepatocellular carcinoma cells in vitro and xenograft tumor formation from these cells in vivo, whereas ABH3 overexpression gave opposite results.

In their work, Shimada et al. demonstrated that ABH3 is an upstream molecule of NOX2 and Tweak induction, which are proteins associated with inflammation, apoptosis, cell growth, and angiogenesis in various tumors [131].

As is the case for ABH2, elevated activity of ABH3, or at least its increased amount, seems to be participate in the pathogenesis and worsening of the course of several cancer types. As mentioned above, researchers are already working to find inhibitors of ABH3, and this work is bearing fruit in terms of inhibiting proliferation of cancer cells [128,129]. Yet the participation of ABH2 and ABH3 in triggering cancer remains elusive. It is still necessary to understand how the activity of these dioxygenases is involved in pathological processes.

### 4.3. ABH4

Unlike many other human dioxygenases from the AlkB family, ABH4 is reported to be able to modify protein substrates [132,133]. For example, Li et al. have demonstrated that ABH4 regulates the demethylation of monomethylated lysine-84 (K84met) in actin [133]. Not so long ago, data were also obtained suggesting that ABH4 can oxidize m^6^A in murine dsDNA [134].

Although substrate specificity of ABH4 remains poorly understood, studies in the last few years revealed its extremely important role in the body of animals. Deletion of ABH4 is embryonically lethal in mice [133]. Additionally, Nilsen et al. have demonstrated that ABH4 depletion in mice leads to spermatogenic defects [135]. In *Danio rerio* oocytes, the level of *Abh4* mRNA is significantly higher than mRNA levels of other dioxygenases of the AlkB family; furthermore, the deletion of *Abh4* leads to considerable defects in epiboly during embryo gastrulation in zebrafish [136]. Accordingly, more and more researchers associate ABH4 with preimplantation development [137].

It is also worth mentioning the work of Yu et al., who, on the basis of quantitative proteomic data, have hypothesized that ABH4 plays some role in the modulation of cytosine methylation in DNA by regulating the expression level of DNMT1 [138].

Recently, ABH4 was found to be overexpressed in adenocarcinoma cells compared to surrounding normal tissues [139]. By quantitative PCR and immunohistochemistry, Peng et al. [140] have revealed that expression levels of ABH1–4 and ABH7 are noticeably elevated in hepatocellular carcinoma tissues compared to normal tissues. Furthermore, high expression levels of ABH4 negatively correlated with overall survival and recurrence-free survival in patients with hepatocellular carcinoma. Overexpression of ABH4 has also been associated with the cancer stage in patients with hepatocellular carcinoma [140].

The expression of ABH4 is higher in non-small cell lung cancer cells compared to the surrounding tissues [141]. In the article just cited, a knockdown of ABH4 caused significant suppression of proliferation of non-small cell lung cancer cells via cell cycle arrest in the G1 phase during the growth of the tumor in vivo. The knockdown of ABH4 suppressed transcription factor E2F1 and expression of its target gene in non-small cell lung cancer cells. Expression levels of ABH4 and E2F1 significantly correlated in clinical samples of non-small cell lung cancer. Moreover, patients with high expression of ABH4 in the tumor had a poor prognosis, suggesting that ABH4 plays a key part in the tumorigenesis of non-small cell lung cancer [141].

In the meantime, an analysis of possible epigenetic factors that may be involved in the progression of epithelial–mesenchymal transition has allowed researchers to identify *ABH4* as a candidate gene that suppresses epithelial–mesenchymal transition [142]. It was also shown in that paper that in patients with colorectal cancer, ABH4 is downregulated. Moreover, a decrease in the amount of ABH4 correlated with metastasis and a poor prognosis in patients with colorectal cancer. It was also found in that work that overexpression of ABH4 suppresses the invasive ability of colorectal cancer cells in vitro as well as their metastatic potential in vivo. Evidently, such a suppressive function of ABH4 is linked with its competitive binding to WDR5 (a key component of the histone–methyltransferase complex) and consequently with a decrease in the amount of histone modification H3K4me3 in target genes, including *MIR21* [142].

The controversial role of ABH4 in different types of cancer raises new questions regarding this family of enzymes. It is important to obtain more knowledge about substrate specificity of ABH4 and its ability to interact with other proteins in order to shed light on the complicated nature of its activity.

### 4.4. ABH5

Dioxygenase ABH5 is localized to cell nuclei. Unlike other human AlkB homologs, ABH5 has at its N terminus an additional alanine-rich sequence and a coiled-coil structure (Figure 3). It has been suggested that these motifs are important for its localization [76]. Of note, the expression of ABH5 in cells is induced by hypoxia via HIF-1α, and this property is unique among known members of the ABH family, implying a specific function of ABH5 in the hypoxia response [143]. ABH5 removes m^6^A from ssDNA and RNA, with the processing of the DNA substrate taking place approximately one and a half times faster than that of RNA [143,144,145,146,147]. In addition, recent evidence indicates that ABH5 also has weak activity toward m^3^C in ssDNA [148]. The biological purpose of this activity remains unclear, but hypothetically, ABH5 can act as an auxiliary repair dioxygenase on ssDNA.

Ensfelder et al. have demonstrated that dioxygenase ABH5 can remove from RNA not only m^6^A but also adenosine doubly methylated at the sixth position (m^6,6^A), which in the vast majority of cases is situated in a conserved sequence of RNA in small ribosomal subunits [149]. Notably, judging by the results obtained in the study just cited, this enzyme needs to release the product from its active center after the first oxidation of the methyl group, possibly in order to load another molecule of αKG into the active center [149].

A comparison of crystal structure between ABH2 and ABH5 has uncovered the reason why ABH5 is active only on single-stranded substrates. In the structure of ABH5, aside from the absence of the loops utilized by ABH2 for interaction with the strand complementary to a damaged DNA strand, there is also a rigid loop that would cause steric hindrance during the binding to dsDNA [146].

*Abh5*-deficient male mice possess elevated amounts of m^6^A in mRNA and are characterized by impaired fertility as a consequence of apoptosis that affects meiotic metaphase spermatocytes [147]. Tang et al. have also stated that the removal of m^6^A from mRNA via the ABH5 activity plays a major part in spermiogenesis [150].

Du et al. [151] have analyzed the expression profile of ABH5 in a developing mouse brain and noticed that the enzyme is ubiquitously expressed in mouse brain tissues, with relatively high levels in the cerebellum and olfactory bulb. Moreover, ABH5 turned out to be mainly colocalized with neuronal marker NeuN, indicating that ABH5 is predominantly expressed in neurons. Of note, the amount of the ABH5 protein dramatically diminished during brain development [151].

In ABH5 knockdown cells, elevated levels of poly(A) mRNA have been detected in the nucleus, suggesting that ABH5 affects the expression of protein regulators of mRNA export [152].

A connection of ABH5 with oxidative stress has been documented too. Apparently, exposure to reactive oxygen species can significantly increase the amount of m^6^A in mRNA through inhibition of ABH5 activity by sumoylation [153].

Recently, ABH5 was also shown to play an important role in the modulation of CD4^+^ T-cell function during experimental autoimmune encephalomyelitis [154].

There are few data in the literature about a significant influence of ABH5 polymorphisms on the development of various human diseases. For example, Ren et al. have detected only a weak effect of 3′-UTR variants rs1378602 and rs8400 of the *ABH5* gene on the risk of hepatoblastoma [155]. On the other hand, it has been determined elsewhere that the rs8400 polymorphism affects negative regulation of ABH5 expression by microRNA miR-186-3p. Namely, the G-to-A substitution caused by the rs8400 polymorphism reduced the ability of miR-186-3p to bind the 3′-UTR of *ABH5* mRNA, thereby leading to elevated expression of ABH5 in neuroblastoma cells [156]. A recent article points to a hypothetical association of some *ABH5* SNPs located in the intronic part of the gene with the development of an autoimmune thyroid disease [157].

At the same time, in the last five years, the number of studies devoted to the role of the ABH5 expression level in various cancers and other diseases is estimated at dozens per year. Upregulation of ABH5 drives uncontrolled activity of genes, playing a critical part in the cell cycle, epithelial–mesenchymal transition, and angiogenesis, thus ultimately leading to aberrant progression of the cell cycle, evasion of apoptosis, and tumor progression [158].

For instance, ABH5 is overexpressed in lung adenocarcinoma cells during intermittent hypoxia [159], in glioblastoma stem-like cells [160,161], and esophageal squamous cell carcinoma cells [162]. In this context, ABH5 overexpression is always linked with a diminished total level of m^6^A in mRNA of these cells and tissues. Conversely, the suppression of ABH5 expression causes significant inhibition of tumor cell proliferation and invasion.

Evidently, the mechanism that underlies this process involves the demethylation of certain targets by ABH5. Zhang et al. [161] have reported that ABH5 demethylates nascent transcripts of transcription factor FOXM1, thereby upregulating its expression. FOXM1 plays a key part in the transition from the G1/S phase to the G2/M phase and in the progression of the M phase of the cell cycle. Additionally, long noncoding RNA antisense to *FOXM1* (FOXM1-AS) promotes the interaction of ABH5 with nascent transcripts of *FOXM1*. The depletion of ABH5 and FOXM1-AS impairs carcinogenesis in glioblastoma stem-like cells through the FOXM1 axis [161].

Zhang et al. have shown that HIF-dependent expression of ABH5 mediates an increase in the percentage of breast cancer stem cells in a breast tumor microenvironment during hypoxia [163]. Han et al. [164] have later confirmed that ABH5 is required for breast tumor growth. Furthermore, their work suggests that protein arginine methyltransferase 6 (PRMT6) directly methylates ABH5 at R283, thus leading to activation of this dioxygenase and subsequently promoting breast tumor growth [164].

Aside from its participation in carcinogenesis, ABH5 also serves as a negative regulator of postischemic angiogenesis via modification of m^6^A and influences blood flow restoration and postischemic angiogenesis in mice with hindlimb ischemia [165]. In patients with recurrent miscarriage, significantly higher expression of ABH5 has been observed and accordingly a decrease in the global level of m^6^A in mRNAs within placental villous tissue, thus pointing to an important role of ABH5 in the pathogenesis of this disease [166].

Nevertheless, there is also a body of evidence indicating involvement of ABH5 downregulation in the pathogenesis of cancer. For instance, decreased ABH5 expression levels are an independent prognostic factor of worse survival in patients with hepatocellular carcinoma [167] and correlate with a worse prognosis in patients with bladder cancer [168]. Yuan et al. [169] have also reported decreased ABH5 levels (correlating with elevated m^6^A amounts) in osteosarcoma cells compared with normal osteoblasts. Overexpression of ABH5 significantly suppressed osteosarcoma cell growth, migration, and invasion and triggered apoptosis of these cells; by contrast, inhibition of ABH5 had opposite effects [169]. Of note, in the case of bladder cancer and osteosarcoma, ABH5 overexpression significantly suppresses the growth, migration, and invasiveness of tumor cells [168,169].

In addition, there is evidence that ABH5 and FTO are important risk factors of rheumatoid arthritis. Luo et al. have examined mRNA levels of *ABH5* and *FTO* and noticed that the mRNA expression of these enzymes in patients with rheumatoid arthritis is significantly reduced compared to a control group; meanwhile, in patients who receive regular treatment, the level of ABH5 mRNA significantly went up [170].

In the last decade, especially from 2019 to 2023, owing to the demonstration of the extensive role of ABH5 in the progression of various types of cancer, this enzyme has become quite popular among scientists as a target for the creation of inhibitors [171,172]. Yet the functions of this dioxygenase in different types of cancers seem to be opposite. The mechanisms underlying the participation of ABH5 in the pathogenesis of different diseases are supposedly linked with modification of m^6^A in different RNA targets. Thus, it appears that we need to learn more about these targets and their involvement in cell functioning.

### 4.5. ABH6

ABH6, which is located in the nucleus and cytoplasm, is widespread among tissues, and its highest expression is observed in the pancreas and testes [81,173]. Huong et al. have reported that *Arabidopsis* Abh6, which is 36% identical to human ABH6, can affect seed germination and survival under abiotic stress, by serving as a possible RNA methylation eraser protein [174].

In their 2022 work, other authors deciphered the structure of holo-ABH6 and of its complexes with ligands [175]. It was shown there that unlike all other dioxygenases of the AlkB family, ABH6 can bind Tris in its active site, in the region where αKG usually binds. According to the results of that work, ABH6 prefers to bind to ssDNA and RNA. The enzyme possesses three unique loop motifs—Flip1, Flip2, and Flip3—which, judging by crystallographic data, are also crucial for the binding of the enzyme to single-stranded substrates. It is relevant to mention that according to the COSMIC database, many mutations implicated in cancer are localized to these regions. At present, however, there are very few findings in the literature on the structure, protein interaction partners, substrates, and activity of human ABH6. The role of ABH6 in human organism also remains to be established.

In a recent research article, the usefulness of ABH6 against alkylating lesions was tested using an *E. coli* strain deficient in AlkB [176]. ABH6 was found to compensate for the AlkB deficiency and to enhance cell resistance to treatment with alkylating agents. Furthermore, a loss of ABH6 in human pancreatic cancer cells increases DNA damage induced by alkylating agents and significantly reduces cancer cell survival.

ABH6 is the most mysterious and understudied human homolog of AlkB. A few existing data imply its participation in the removal of methylation-induced lesions from single-stranded nucleic acids. Nevertheless, its impact in this process as compared to other AlkB-like enzymes such as ABH1, ABH3, and ABH5 remains unclear. One of possible reasons why this enzyme is so poorly understood is its ability to bind Tris in its active center, considering that this is a popular buffer component. Choosing different reaction conditions could shed light on ABH6’s substrate specificity. It is fair to say that there is an open field for research on ABH6.

### 4.6. ABH7

Murine ABH7 is localized to the mitochondrial matrix [177]. The crystal structure of ABH7 has shown that, unlike other members of the AlkB family whose substrates are DNA or RNA, ABH7 is devoid of the “nucleotide recognition lid”, which is required for nucleotide binding, and therefore has a solvent-exposed active site; in the ABH7 protein, certain structures form a negatively charged groove. These distinctive features have led to the supposition that ABH7 interacts with protein substrates rather than nucleic acids [178].

Despite these earlier conclusions, Zhang et al., also after a comparison of crystal structures between ABH7 and AlkB, have proposed an RNA demethylase function for this enzyme [179]. Moreover, those authors were able to experimentally confirm that ABH7 can indeed remove *N*^2^-dimethylguanosine (m^2,2^G) and m^1^A from nascent mitochondrial RNA. Suppression of ABH7 expression resulted in lowered levels of mitochondrial RNA and of mitochondrial proteins, and the two phenomena together diminished mitochondrial activity. In mouse adipose tissue, the loss of ABH7 impairs the functioning of oxidative phosphorylation and reduces the fatty acid oxidation activity, thus inducing fat accumulation and an obese phenotype [179].

In the first work on the functional characterization of murine *Abh7*, it was also noted that this gene’s deletion causes a significant increase in body weight and fat percentage in mice [177]. Hence, it can be concluded that ABH7 plays an important role in fatty acid metabolism through its RNA demethylase activity.

Studies on cell lines have shown that *Abh7* deletion suppresses necrotic cell death induced by treatment with alkylating and oxidizing agents, while not affecting apoptotic cell death [84]. This observation indicates that ABH7 participates in protection from alkylating damage along with other enzymes of the family.

In their targeted search for the SNPs in the AlkB family that are associated with prostate cancer, Walker et al. identified an SNP of *ABH7* (rs7540) that significantly correlates with prostate cancer in two separate cohorts [180]. Their comparison of molecular dynamics simulations between the wild-type and mutant protein structures suggested that the SNP-caused substitution R191Q in ABH7 causes substantial structural alterations in this protein that reduce its ability to bind its cofactor Fe(II) and cosubstrate αKG. Experimental spectroscopic analyses of purified proteins confirmed the predictions of the molecular dynamics simulations [180].

Considering the supposed participation of ABH7 in the maintenance of the methylation of mitochondrial RNA, consequences of the impairment of the ABH7 activity should extend much more widely. Thus, it is important to guide research in this direction by studying changes in expression of ABH7 and association of its possible SNPs with various pathological conditions. It is also important to deeply investigate substrate specificity of ABH7 in order to learn about its possible nucleic acid and protein targets.

### 4.7. ABH8

The ABH8 enzyme is somewhat unique as compared to other human homologs of AlkB, because aside from the dioxygenase domain, this enzyme contains a methyltransferase domain (MTase, Figure 3) homologous to yeast tRNA methyltransferase Trm9p [181,182]. An SAM-dependent MTase subdomain of ABH8 is located in the C-terminal part of the AlkB-type oxygenase domain; ABH8 also possesses supplementary RNA recognition motifs (RRMs) at the N terminus [183,184]. Fu et al. have found [183] that ABH8 catalyzes tRNA methylation thus creating 5-methylcarboxymethyluridine (mcm^5^U) at the wobble position in some tRNAs: this is a critical modification of the anticodon loop. Deletion of ABH8 in human cells lowers endogenous amounts of mcm^5^U in RNA and enhances the sensitivity of the cells to DNA-damaging agents. Moreover, DNA-damaging agents induce the expression of ABH8 in an ATM-dependent manner [85,183,185,186]. Furthermore, at the time, no demethylase activity of ABH8 has been detected [185].

In vivo experiments on *Drosophila* suggest that ABH8 participates in the control of oxidative stress in the brain while inhibiting synaptic growth and supporting learning and memory [187]. According to that paper, in ABH8-null animals, uridine methylation at the wobble position is absent, and the animals exhibit a global reduction in protein synthesis, including a specific decline in selenoprotein levels. The loss of ABH8 or independent impairment of selenoprotein synthesis resulted in ectopic synapse formation. Forced expression of antioxidant enzymes completely suppressed the excessive synaptic growth in the ABH8-null animals, implying that oxidative stress is the underlying cause of this dysregulation. In that work, ABH8-null animals also exhibited impairments in associative learning and memory, which were rescued by pharmacological treatment with antioxidants. Taken together, these findings mean a critical function of tRNA modifications in redox homeostasis within the nervous system and suggest that antioxidants may be administered as a potential therapy for ABH8-associated intellectual disability [187].

In two multiplex consanguineous families, Monies et al. [188] have identified two homozygous truncation mutants of ABH8 that cause intellectual disability. An analysis of tRNA from the affected individuals revealed a complete absence of mcm^5^U, consistently with the predicted loss of function of the mutant enzymes. These findings indicate the sensitivity of brain tissue to modifications at the tRNA wobble position and expand the list of mental retardation syndromes caused by mutations in genes associated with tRNA modifications [188]. Later, Saad et al. [189] have described another family of Egyptian origin carrying a novel homozygous frameshift variant in the last exon of *ABH8*. Several members of this family show a global developmental delay and some dysmorphic features of appearance [189].

Also recently, in one of patients with developmental delay, an ABH8 mutant was identified in which a missense mutation creates substitution R625H [190]. Later, an ABH8 mutant carrying substitution R625P was detected in Turkish patients with similar symptoms [191]. Arg625 is a highly conserved residue in ABH8 from unicellular eukaryotes to humans.

In another patient with developmental delay, in the *ABH8* gene, a biallelic missense variant was found that leads to a mutant enzyme carrying substitution W504S [192]. The identified substitution is situated in the highly conserved tRNA MTase domain. The patient carrying the mutant enzyme exhibited intellectual disability, facial dysmorphism, speech delay, and learning disabilities, which are common features among the patients reported by Monies et al. [188]. Taken together, these observations suggest that *ABH8* is a gene with a recently discovered important role in nervous system diseases.

Dioxygenase ABH8 is also implicated in resistance to programmed cell death in human urothelial carcinoma cells, thus eventually leading to bladder cancer. This enzyme has been shown to be overexpressed in bladder cancer tissues, and its knockdown induces apoptosis in this tumor cell type [182]. The mechanism driving this process seems to include downregulation of the survivin protein, an antiapoptotic factor that also shows elevated levels in bladder cancer [193].

Altogether, these data indicate a high importance of ABH8 in the pathogenesis of various diseases, especially nervous system diseases. These data make this enzyme a promising target for medical treatments. Indeed, there are reports that ABH8 is already used as a target in some types of antitumor therapy [182,194]. The example of ABH8 is particularly interesting because of its unique methyltransferase activity. It is possible that in the connection of this activity with a demethylase activity (which has not yet been found in ABH8), there are hidden nuances of fine-tuning mechanisms involving other AlkB-like human dioxygenases in several physiological processes.

### 4.8. FTO

Although FTO was identified primarily as a candidate gene associated with obesity risk, it has been predicted bioinformatically to be an Fe(II)/αKG-dependent dioxygenase homologous to bacterial DNA dioxygenase AlkB [80]. A recombinant FTO protein can remove methyl groups from such lesions as m^3^T, 3-methyluracil (m^3^U), and m^6^A in ssDNA and RNA [80,195,196]. m^6^A is the most widespread modified nucleoside found in mRNA [197], and it is demethylated by FTO 50-fold more intensively than m^3^U is [196], which is located primarily in ribosomal RNA [198]. Nonetheless, because most of the total RNA pool in the cell consists of rRNA, in absolute amounts there is actually ~100-fold more m^3^U than m^6^A in the cell [199].

Lately, it has been demonstrated that FTO has certain selectivity in terms of binding to and removing m^6^A from m^6^A motifs, thus confirming FTO’s possible functioning in the regulation of the dynamics and distribution of the m^6^A/m^6,6^A mark in various physiological and pathological conditions [200].

Crystal structure of FTO has been deciphered: this protein possesses an N-terminal catalytic domain and a C-terminal domain of an unknown function [87]. The catalytic pocket contains five aas that are conserved among all members of this enzymatic superfamily: two residues, His and Asp, are required for the binding of Fe(II), and three residues—histidine and two arginines (separated by six aas)—are required for the binding of αKG [87,201]. Specificity for single-stranded nucleic acids is ensured by loop L1 (absent in other members of the AlkB family), which sterically hinders the entry of double-stranded nucleic acids into the catalytic pocket [87]. A study on the dynamics and structure of human FTO in solution indicates that the structure of the catalytic N-terminal domain is unstable in the absence of the C-terminal domain. This observation explains the absence of activity in the isolated N-terminal domain and suggests that the interaction of the domains may be a good target for the design of specific inhibitors [86].

Notably, although FTO is present in all animal tissues, including human ones [80], the highest expression of this protein is seen in the brain, including the hypothalamus [80], where the center controlling food intake is situated [202].

Although many research articles have mainly addressed the role of DNA modifications in the formation of long-term memory, Walters et al. [203] have highlighted the contribution of RNA modifications to this process. *Fto* is expressed in the nuclei, dendrites, and peridendritic spines of neurons in the CA1 area of the murine dorsal hippocampus. In that article, contextual fear conditioning for short periods of time reduced FTO levels in these neurons, and the greatest downregulation of FTO was registered near synapses. Artificial depletion of FTO in the dorsal hippocampus of normal (wild-type) mice by microinjection of several types of *Fto*-targeting vectors caused significant enhancement of contextual fear memory. Taken together, these results point to the importance of FTO during memory formation and imply that mRNA modifications and epitranscriptomics are novel regulators of memory formation [203].

Cao et al., by means of a conditional knockout in mice, have demonstrated that specific ablation of *Fto* in adult neural stem cells transiently accelerates their proliferation and promotes neuronal differentiation both in vitro and in vivo, but in the long term, the specific ablation of *Fto* inhibited neurogenesis and neuronal development in adult individuals [204]. Furthermore, FTO expression generally declines with age, as proven for mouse follicular fluid, granulosa cells, and ovaries [205].

There is also evidence of a possible link between *Fto* expression and depressive behavior in mice [206]. In a study by Liu et al. [206], a decrease in *Fto* expression in the hippocampus induced behavior characterized as depressive (depression-like). Conversely, *Fto* overexpression reversed the depression-like phenotype.

FTO partakes in the conversion of white adipose tissue to brown adipose tissue. For example, Wu et al. have reported that a loss of FTO promotes the expression of thermogenic genes through an increase in the levels of m^6^A in mRNA and of the HIF1A protein [207]. Zhang et al. have revealed that FTO participates in preadipocyte differentiation as well, via demethylation of m^6^A in RNA [208].

An FTO knockdown by means of small interfering RNA gives an overall increase in m^6^A levels in RNA as well as elevated expression of YTH domain family member 2, which binds to m^6^A [209]. Additionally, the same researchers noticed that the FTO knockdown significantly diminishes levels of de novo lipogenic enzymes and the intracellular lipid content, through an increase in the amounts of m^6^A on the mRNAs that are involved in these processes.

Wang et al. [210] have determined that FTO expression goes up during myoblast differentiation, whereas FTO silencing inhibits this differentiation; furthermore, skeletal muscle development is impaired in mice deficient in FTO within skeletal muscle. Notably, myogenic differentiation stimulated by FTO was dependent on its m^6^A-demethylating activity [210].

The best-known and best-studied variant of FTO is intronic polymorphism rs9939609. There is a fairly large body of data linking this SNP rs9939609 with a higher risk of obesity [211]. Of note, many researchers in the last few years associated rs9939609 in particular and FTO overexpression in general with dietary habit changes that are responsible for the development of obesity [212,213]. A small study implies a link between rs9930506 and a predisposition to obesity in Greek adults [214].

In the meantime, studies on mouse models have shown that substitution I367F in murine FTO yields a phenotype with reduced body weight and fat mass, apparently due to an increase in metabolic rate [215]. In *Fto* knockout mice, a phenotype is observed that is characterized by delayed postnatal growth, decreased fat and muscle mass, and elevated food intake (when the data are adjusted for muscle mass) [216]. All this is accompanied by a substantial rate of postnatal mortality: only 50% of homozygous pups survive to weaning [216,217].

In humans, the FTO SNP that causes substitution R316Q and a loss of FTO activity gives an even more complex phenotype: postnatal growth retardation, microcephaly, severe psychomotor retardation, functional brain disorders, and a characteristic facial dysmorphism [218]. Amino acid residue Arg316 takes part in the coordination of αKG in the active site of FTO and is absolutely conserved among all FTO paralogs and AlkB orthologs [218]. Later, several more deleterious SNPs were identified that lead to aa substitutions (in the same functional domain where Arg316 does) and to a loss of activity in FTO. For example, a homozygous missense mutation creating substitution S319F has been identified in a neonate with growth retardation and severe developmental delay [219]; in patients from a consanguineous Yemeni family, an FTO variant has been identified that carries substitution R322Q and is associated with a lethal birth defect syndrome involving eye anomalies, gingival overgrowth, craniosynostosis, and cutaneous photosensitivity [220].

*FTO* SNP rs62033438, located in the intronic region of the gene, correlates with male infertility according to Landfors et al. [221]. In addition, the same work revealed two more potentially deleterious missense mutations in *FTO* that give substitutions C326S and S256N in the protein. Cys326 is localized to an important linker between the two protein domains of FTO, whereas Ser256 is within a flexible loop capable of interacting with other molecules [221].

Mayman et al. [222] reported the first FTO variant containing an aa substitution that is outside the catalytic site but causes multiple abnormalities in multiple organ systems, thus affecting respiratory, cardiovascular, and neurological functions. In that paper, a ≤90% loss of demethylase activity was demonstrated in vitro in the FTO variant R96P compared to the wild-type enzyme [222].

FTO is upregulated in many cancers, and its high expression correlates with lower overall survival [7]. For example, FTO is frequently overexpressed in tissues of cervical cancer [223] and of human breast cancer [224]. The inhibition of FTO suppresses melanoma cell tumorigenicity and expression of melanoma cell-intrinsic genes, including *PD1*; this treatment sensitizes melanoma cells to anti-PD1 therapy in mice [225].

Liu et al. have established that FTO is an important epitranscriptomic regulator used by tumors to evade immune surveillance through modulation of glycolytic metabolism [226]. In the research article just cited, FTO-mediated demethylation of m^6^A in tumor cells upregulated transcription factors c-Jun, JunB, and C/EBPB, thereby allowing cells to rewire their glycolytic metabolism. An FTO knockdown disturbed the glycolytic activity of tumor cells, thus restoring the function of CD8^+^ T cells, thereby inhibiting tumor growth.

On the other hand, a bioinformatic study supported by experimental validation in 30 cases indicates that FTO expression is low in thyroid cancer tissues and correlates with lymph node metastasis in thyroid cancer patients [227].

Aside from the association of FTO with the progression of many cancers [7], obesity [211,228,229,230], and type 2 diabetes mellitus [231,232], some investigators link this enzyme with Alzheimer’s disease [233,234] and nonalcoholic steatohepatitis too [235]. In this regard, much attention of researchers is focused on the development of inhibitors of this enzyme.

For instance, *N*-oxalylglycine (a dioxygenase inhibitor competing with αKG for binding to the protein) is already widely used in crystallographic analyses to prepare stable enzyme–substrate complexes as well as in functional studies on Fe(II)/αKG-dependent dioxygenases, for example, to assess the involvement of FTO in epigenetic regulation of genes [96,236,237]. In cellular and murine models, investigation into the inhibition of FTO by a derivative of meclofenamic acid (which is an inhibitor that associates with a nucleic acid substrate binding site) suggests that FTO suppression by selective inhibitors may be a successful strategy for the treatment of acute myeloid leukemia [96,236].

## 5. Summary and Perspectives

The last couple of decades of research have greatly expanded our understanding of the diverse and distinct functions of human AlkB homologs. Although all nine enzymes apparently derive from a common ancestor, their large number is justified by substantial differences in their substrate specificity, localization, and functions in the cell (Table 1) as well as consequences of changes in the expression levels of these enzymes, their disfunctions, or SNP effects (Table 2).

Although there is increasing evidence in the modern literature supporting the function of m^6^A in DNA as a new epigenetic mark, there are still very few data on the only known enzyme capable of removing it: ABH1. Aside from the scant information about substrate specificity of this dioxygenase, which is the closest to AlkB among all human homologs, there are also very few data in the literature on its role in the human organism and on consequences of its malfunction.

Meanwhile, the growing understanding of the enormous importance of m^6^A in RNA has drawn the attention of researchers to dioxygenases ABH5 and FTO. Both have been shown to be expressed in neurons. For both, one of the main targets is m^6^A in RNA. Moreover, the fact that a malfunction of one of these enzymes can be detected relatively independently of the presence of the other allows us to conclude that in the human body, functions and individual targets of these enzymes are somehow separated. Numerous studies, hundreds and thousands, have addressed the involvement of ABH5 and FTO in various types of cancer, with results that are not so clear-cut. It seems that FTO has a primary role not only in fat mass regulation but also in brain development, in contrast to ABH5, although this enzyme is also located in neurons. Moreover, a large body of data on developmental effects of FTO SNPs and almost complete absence of data on ABH5 SNPs raise new questions. Has not enough attention been paid to this topic regarding ABH5, or is the function of this enzyme too important to detect its weakened variants in living individuals? Furthermore, one should not forget the deleterious effect of both decreased FTO activity due to mutations and its overexpression in many cancers along with ABH5 disturbances.

Another dioxygenase that has been found to play a major role in brain development and function is dioxygenase ABH8. This enzyme is unique among all other human AlkB homologs because it performs an important function by means of its methylating activity, although its demethylating activity has not been demonstrated yet. It would be intriguing to study this enzyme in the context of a relation between its methylating and demethylating activities or to explain the absence of the latter.

ABH2 and ABH3 can be called the main dioxygenases protecting genomic DNA from methyl lesions such as m^1^A and m^3^C. Of note, the existing body of evidence indicates that enhanced activity of the two dioxygenases significantly worsens the prognosis of patients with certain types of cancer and is generally observed in cancer cells. On the other hand, little is known about the existing SNPs of these enzymes, except for a couple of SNPs in *ABH2*. Yet the roles of ABH2 and ABH3 in carcinogenesis remain elusive. It is still necessary to understand how the activity of these dioxygenases is involved in pathological processes.

There are a lot of studies in the literature about substrate specificity and its mechanisms for enzymes ABH2 and ABH3. Nonetheless, this knowledge does not provide a complete picture elucidating their functions in the human body. At the same time, paradoxically, for the ABH4 enzyme, whose substrate specificity remains extremely poorly investigated, its substantial role in the organism has been demonstrated for many processes. At the same time, the mechanisms underlying this role remain poorly understood. Generally, the controversial participation of ABH4 in different types of cancer raises new questions regarding this family of enzymes.

ABH6 remains the least studied human homolog of AlkB. Its involvement in the removal of methylation-induced lesions from single-stranded nucleic acids—in contrast to other homologs such as ABH1, ABH3, and ABH5—remains elusive. Recent reports proving the ability of this enzyme to bind Tris in its active site provide hope that a change in the approach to studying ABH6’s enzymatic activity may soon yield a better understanding of this enzyme’s substrate specificity and function.

There is also very little information in the literature on ABH7. Given a supposed role of this enzyme in the removal of methyl damage from mitochondrial DNA, it can be assumed that disturbances in its functioning can lead to serious consequences for the organism. Therefore, further research into its functional properties and participation in various physiological processes seems extremely important.

To summarize the discussion presented here, the unique differences between the nine human homologs of AlkB enzymes still intrigue researchers. Despite the enormous efforts devoted to studying some of these enzymes, others remain in the shadows. There is an open field for research into the AlkB family of enzymes regarding their functions in the human organism, including through the detection and investigation of cases of their malfunction, particularly in relation to the emergence of various mutations.

## 6. Conclusions

In this review, up-to-date information about eukaryotic representatives of the family of AlkB-like nonheme Fe(II)/α-ketoglutarate-dependent dioxygenases responsible for the dealkylation of macromolecules in the cell is discussed. The reader is introduced to the main types of methylated adducts that arise in nucleic acids as a result of the impact of endogenous and exogenous factors. The main body of the review is dedicated to modern knowledge about the functional characteristics and biological roles of AlkB-like human homologues in dealkylation processes. Specific features of protein structure, substrate specificity, known role in the organism, and known consequences of disruption of these enzymes’ functioning are discussed for each AlkB human homolog. Special attention is given to reports about effects of natural single-nucleotide polymorphisms on the activity of these enzymes and to potential consequences for carriers of such natural variants.

## Figures and Tables

**Table 1:** Substrate specificity and functions of human AlkB homologs.

Enzyme	Substrates	Functionality	References
ABH1	m^3^C in RNA/ssDNA (weak activity), m^6^A in ssDNA, m^1^A in tRNA, m^5^C in RNA	Demethylation of m^6^A in DNA: another important epigenetic mark; role in spermatogenesis and embryonic development (in mice); modification of mitochondrial tRNAs.	[47,52,55,92,93,94,95,96,97,98,100]
ABH2	m^1^A, m^3^C, m^5^C, εA in dsDNA	Important for transcription and integrity of rDNA.	[111,113,117]
ABH3	m^1^A, m^3^C, m^5^C, εA in ssDNA; m^1^A, m^1,6^A, m^3^C in tRNA	Removal of m^1^A from RNA; promotion of proliferation, migration, and invasiveness of tumor cells.	[79,112,113,116,118,119]
ABH4	monomethylated lysine-84 in actin; m^6^A in dsDNA	Spermatogenesis, embryogenesis (mice); embryogenesis (fish); proliferation of cancer cells.	[133,134,135,136,141]
ABH5	m^6^A in ssDNA and RNA; m^3^C in ssDNA (weak activity); m^6,6^A in RNA	Expression of ABH5 in cells is induced by hypoxia via HIF-1α; male fertility (mice) and spermiogenesis; predominantly expressed in neurons; cell cycle, epithelial–mesenchymal transition, and angiogenesis.	[143,144,145,146,147,148,149,150,151,158]
ABH6	unknown	Unknown	
ABH7	m^2,2^G, m^1^A in mitochondrial RNA; proteins?	Fatty acid metabolism.	[177,178,179]
ABH8	Methylation of 5-methylcarboxyuridine in tRNA	Control of oxidative stress in the brain (fly); brain development.	[183,187,188,189]
FTO	m^3^T, m^3^U, m^6^A in ssDNA and RNA	Memory formation; possible role in depression; conversion of white adipose tissue to brown adipose tissue; myoblast differentiation; epitranscriptomic regulator.	[80,195,196,203,206,207,210,226]

**Table 2:** Effects of changes in the expression level or of SNPs of human AlkB homologs.

Enzyme	Changes in Expression Level or SNPs	Effect	References
ABH1	rs2267755 (3′-UTR C>T)	Reduces the risk of neuroblastoma.	[107]
	Overexpression	Poor outcomes in gastric adenocarcinoma.	[104]
		Registered in hepatocellular carcinoma.	[140]
ABH2	I141V (highly conserved residue)	Found in glioma.	[120]
	A9V, Q10K (PCNA-binding region)	Cancer-associated.	[121]
	Overexpression	Registered in hepatocellular carcinoma.	[140]
ABH3	Overexpression	Registered in hepatocellular carcinoma.	[130,140]
ABH4	Overexpression	Registered in adenocarcinoma.	[139]
		Registered in hepatocellular carcinoma.	[140]
		Registered in non-small cell lung cancer.	[141]
	Downregulation	Registered in colorectal cancer.	[142]
ABH5	rs137860, rs8400 * (3′-UTR variants)	Risk of hepatoblastoma (weak effect).	[155]
	Overexpression	Registered in lung adenocarcinoma.	[159]
		Registered in glioblastoma stem-like cells	[160,161]
		Registered in esophageal squamous cell carcinoma.	[162]
		Associated with recurrent miscarriage.	[166]
	Downregulation	Associated with rheumatoid arthritis.	[170]
		Registered in osteosarcoma.	[169]
ABH6	No data available	-	-
ABH7	R191Q	Correlation with prostate cancer.	[180]
	Overexpression	Registered in hepatocellular carcinoma.	[140]
ABH8	Arg554∗; Trp599Glyfs∗19 (truncated)	Associated with intellectual disability.	[188]
	Frameshift variant in the last exon	Associated with global developmental delay.	[189]
	R625H; R625P (highly conserved residue)	Associated with developmental delay.	[190,191,192]
	W504S (MTase domain)		
	Overexpression	Registered in bladder cancer.	[182]
FTO	rs9939609 (intronic variant)	Higher risk of obesity.	[211,214]
	R316Q (catalytic domain)	Found in cases of postnatal growth retardation, microcephaly, severe psychomotor retardation, functional brain disorders, and a characteristic facial dysmorphism.	[218]
	S319F (catalytic domain)	Found in cases of growth retardation and severe developmental delay.	[219]
	R322Q (catalytic domain)	Found in cases of lethal birth defects.	[220]
	rs62033438 (intronic variant)	Associated with male infertility.	[221]
	S256N; C326S (catalytic domain)	Potentially deleterious.	[221]
	R96P	Found in cases of multiple abnormalities in multiple organ systems, thus affecting respiratory, cardiovascular, and neurological functions.	[222]
	Overexpression	Registered in cervical cancer.	[223]
		Registered in breast cancer.	[224]
		Diet-dependent development of obesity.	[212,213]
	Downregulation	Registered in thyroid cancer.	[227]

* The rs8400 3′-UTR variant of *ABH5* has been demonstrated to lead to the overexpression of ABH5 in neuroblastoma cells [156].

## References

[1] De Bont R. (2004). Endogenous DNA Damage in Humans: A Review of Quantitative Data. Mutagenesis.

[2] Galperin M.Y., Moroz O.V., Wilson K.S., Murzin A.G. (2006). House Cleaning, a Part of Good Housekeeping. Mol. Microbiol..

[3] Lindahl T. (1993). Instability and Decay of the Primary Structure of DNA. Nature.

[4] Sedgwick B., Bates P.A., Paik J., Jacobs S.C., Lindahl T. (2007). Repair of Alkylated DNA: Recent Advances. DNA Repair..

[5] Sall S.O., Johann To Berens P., Molinier J., Jasiulionis M.G. (2022). Chapter 1—DNA Damage and DNA Methylation. Epigenetics and DNA Damage.

[6] Peng Y., Pei H. (2021). DNA Alkylation Lesion Repair: Outcomes and Implications in Cancer Chemotherapy. J. Zhejiang Univ. Sci. B.

[7] Li Q., Zhu Q. (2023). The Role of Demethylase AlkB Homologs in Cancer. Front. Oncol..

[8] Comb M., Goodman H.M. (1990). CpG Methylation Inhibits Proenkephalin Gene Expression and Binding of the Transcription Factor AP-2. Nucleic Acids Res..

[9] Nan X., Ng H.H., Johnson C.A., Laherty C.D., Turner B.M., Eisenman R.N., Bird A. (1998). Transcriptional Repression by the Methyl-CpG-Binding Protein MeCP2 Involves a Histone Deacetylase Complex. Nature.

[10] Smith Z.D., Meissner A. (2013). DNA Methylation: Roles in Mammalian Development. Nat. Rev. Genet..

[11] Buitrago D., Labrador M., Arcon J.P., Lema R., Flores O., Esteve-Codina A., Blanc J., Villegas N., Bellido D., Gut M. (2021). Impact of DNA Methylation on 3D Genome Structure. Nat. Commun..

[12] Pérez A., Castellazzi C.L., Battistini F., Collinet K., Flores O., Deniz O., Ruiz M.L., Torrents D., Eritja R., Soler-López M. (2012). Impact of Methylation on the Physical Properties of DNA. Biophys. J..

[13] Rao S., Chiu T.-P., Kribelbauer J.F., Mann R.S., Bussemaker H.J., Rohs R. (2018). Systematic Prediction of DNA Shape Changes Due to CpG Methylation Explains Epigenetic Effects on Protein–DNA Binding. Epigenetics Chromatin.

[14] Hamidi T., Singh A.K., Chen T. (2015). Genetic Alterations of DNA Methylation Machinery in Human Diseases. Epigenomics.

[15] He P.C., He C. (2021). M6 A RNA Methylation: From Mechanisms to Therapeutic Potential. EMBO J..

[16] Luo C., Hajkova P., Ecker J.R. (2018). Dynamic DNA Methylation: In the Right Place at the Right Time. Science.

[17] Matrisciano F., Dong E., Nicoletti F., Guidotti A. (2018). Epigenetic Alterations in Prenatal Stress Mice as an Endophenotype Model for Schizophrenia: Role of Metabotropic Glutamate 2/3 Receptors. Front. Mol. Neurosci..

[18] Stojković V., Fujimori D.G. (2017). Mutations in RNA Methylating Enzymes in Disease. Curr. Opin. Chem. Biol..

[19] Xie P., Zang L.-Q., Li X.-K., Shu Q. (2016). An Epigenetic View of Developmental Diseases: New Targets, New Therapies. World J. Pediatr..

[20] Mazin A.L. (2009). Suicidal Function of DNA Methylation in Age-Related Genome Disintegration. Ageing Res. Rev..

[21] Hoernes T.P., Clementi N., Faserl K., Glasner H., Breuker K., Lindner H., Hüttenhofer A., Erlacher M.D. (2016). Nucleotide Modifications within Bacterial Messenger RNAs Regulate Their Translation and Are Able to Rewire the Genetic Code. Nucleic Acids Res..

[22] Hudson B.H., Zaher H.S. (2015). O6-Methylguanosine Leads to Position-Dependent Effects on Ribosome Speed and Fidelity. RNA.

[23] Thomas E.N., Kim K.Q., McHugh E.P., Marcinkiewicz T., Zaher H.S. (2020). Alkylative Damage of mRNA Leads to Ribosome Stalling and Rescue by Trans Translation in Bacteria. eLife.

[24] You C., Dai X., Wang Y. (2017). Position-Dependent Effects of Regioisomeric Methylated Adenine and Guanine Ribonucleosides on Translation. Nucleic Acids Res..

[25] Cole S.E., LaRiviere F.J., Merrikh C.N., Moore M.J. (2009). A Convergence of rRNA and mRNA Quality Control Pathways Revealed by Mechanistic Analysis of Nonfunctional rRNA Decay. Mol. Cell.

[26] Nawrot B., Sochacka E., Düchler M. (2011). tRNA Structural and Functional Changes Induced by Oxidative Stress. Cell. Mol. Life Sci..

[27] Willi J., Küpfer P., Evéquoz D., Fernandez G., Katz A., Leumann C., Polacek N. (2018). Oxidative Stress Damages rRNA inside the Ribosome and Differentially Affects the Catalytic Center. Nucleic Acids Res..

[28] Tsao N., Schärer O.D., Mosammaparast N. (2021). The Complexity and Regulation of Repair of Alkylation Damage to Nucleic Acids. Crit. Rev. Biochem. Mol. Biol..

[29] Yan L.L., Zaher H.S. (2019). How Do Cells Cope with RNA Damage and Its Consequences?. J. Biol. Chem..

[30] Goldberg A.L. (2003). Protein Degradation and Protection against Misfolded or Damaged Proteins. Nature.

[31] Grune T., Reinheckel T., Davies K.J.A. (1997). Degradation of Oxidized Proteins in Mammalian Cells. FASEB J..

[32] Vilchez D., Saez I., Dillin A. (2014). The Role of Protein Clearance Mechanisms in Organismal Ageing and Age-Related Diseases. Nat. Commun..

[33] Stadtman E.R., Moskovitz J., Levine R.L. (2003). Oxidation of Methionine Residues of Proteins: Biological Consequences. Antioxid. Redox Signal..

[34] Boschi-Muller S., Gand A., Branlant G. (2008). The Methionine Sulfoxide Reductases: Catalysis and Substrate Specificities. Arch. Biochem. Biophys..

[35] Aletta J.M., Cimato T.R., Ettinger M.J. (1998). Protein Methylation: A Signal Event in Post-Translational Modification. Trends Biochem. Sci..

[36] Comb D.G., Sarkar N., Pinzino C.J. (1966). The Methylation of Lysine Residues in Protein. J. Biol. Chem..

[37] Paik W.K., Kim S. (1968). Protein Methylase I: Purification and Properties of the Enzyme. J. Biol. Chem..

[38] Stanevich V., Jiang L., Satyshur K.A., Li Y., Jeffrey P.D., Li Z., Menden P., Semmelhack M.F., Xing Y. (2011). The Structural Basis for Tight Control of PP2A Methylation and Function by LCMT-1. Mol. Cell.

[39] Cheng D., Vemulapalli V., Bedford M.T., Wu C., Allis C.D. (2012). Chapter Four—Methods Applied to the Study of Protein Arginine Methylation. Methods in Enzymology.

[40] Greer E.L., Shi Y. (2012). Histone Methylation: A Dynamic Mark in Health, Disease and Inheritance. Nat. Rev. Genet..

[41] Rydberg B., Lindahl T. (1982). Nonenzymatic Methylation of DNA by the Intracellular Methyl Group Donor S-Adenosyl-L-Methionine Is a Potentially Mutagenic Reaction. EMBO J..

[42] Lyko F. (2018). The DNA Methyltransferase Family: A Versatile Toolkit for Epigenetic Regulation. Nat. Rev. Genet..

[43] Lawley P.D., Brookes P. (1963). Further Studies on the Alkylation of Nucleic Acids and Their Constituent Nucleotides. Biochem. J..

[44] Bodell W.J., Singer B. (1979). Influence of Hydrogen Bonding in DNA and Polynucleotides on Reaction of Nitrogens and Oxygens toward Ethylnitrosourea. Biochemistry.

[45] Li H., Zhang N., Wang Y., Xia S., Zhu Y., Xing C., Tian X., Du Y. (2022). DNA N6-Methyladenine Modification in Eukaryotic Genome. Front. Genet..

[46] Greer E.L., Blanco M.A., Gu L., Sendinc E., Liu J., Aristizábal-Corrales D., Hsu C.-H., Aravind L., He C., Shi Y. (2015). DNA Methylation on N6-Adenine in *C. elegans*. Cell.

[47] Ma C., Niu R., Huang T., Shao L.-W., Peng Y., Ding W., Wang Y., Jia G., He C., Li C.-Y. (2019). N6-Methyldeoxyadenine Is a Transgenerational Epigenetic Signal for Mitochondrial Stress Adaptation. Nat. Cell Biol..

[48] Liang Z., Shen L., Cui X., Bao S., Geng Y., Yu G., Liang F., Xie S., Lu T., Gu X. (2018). DNA N6-Adenine Methylation in Arabidopsis Thaliana. Dev. Cell.

[49] Shah K., Cao W., Ellison C.E. (2019). Adenine Methylation in Drosophila Is Associated with the Tissue-Specific Expression of Developmental and Regulatory Genes. G3.

[50] Zhang G., Huang H., Liu D., Cheng Y., Liu X., Zhang W., Yin R., Zhang D., Zhang P., Liu J. (2015). N6-Methyladenine DNA Modification in Drosophila. Cell.

[51] Hao Z., Wu T., Cui X., Zhu P., Tan C., Dou X., Hsu K.-W., Lin Y.-T., Peng P.-H., Zhang L.-S. (2020). N6-Deoxyadenosine Methylation in Mammalian Mitochondrial DNA. Mol. Cell.

[52] Wu T.P., Wang T., Seetin M.G., Lai Y., Zhu S., Lin K., Liu Y., Byrum S.D., Mackintosh S.G., Zhong M. (2016). DNA Methylation on N6-Adenine in Mammalian Embryonic Stem Cells. Nature.

[53] Xiao C.-L., Zhu S., He M., Chen D., Zhang Q., Chen Y., Yu G., Liu J., Xie S.-Q., Luo F. (2018). N6-Methyladenine DNA Modification in the Human Genome. Mol. Cell.

[54] Liu J., Zhu Y., Luo G.-Z., Wang X., Yue Y., Wang X., Zong X., Chen K., Yin H., Fu Y. (2016). Abundant DNA 6mA Methylation during Early Embryogenesis of Zebrafish and Pig. Nat. Commun..

[55] Xiong L., Li F., Guo Y., Zhang J., Xu K., Xiong Z., Tong A., Li L., Yang S. (2024). Discovery of a Potent and Cell-Active Inhibitor of DNA 6mA Demethylase ALKBH1. J. Am. Chem. Soc..

[56] Douvlataniotis K., Bensberg M., Lentini A., Gylemo B., Nestor C. (2020). No Evidence for DNA N 6-Methyladenine in Mammals. Sci. Adv..

[57] Koivisto P., Robins P., Lindahl T., Sedgwiek B. (2004). Demethylation of 3-Methylthymine in DNA by Bacterial and Human DNA Dioxygenases. J. Biol. Chem..

[58] Demple B., Sedgwick B., Robins P., Totty N., Waterfield M.D., Lindahl T. (1985). Active Site and Complete Sequence of the Suicidal Methyltransferase That Counters Alkylation Mutagenesis. Proc. Natl. Acad. Sci. USA.

[59] Daniels D.S., Tainer J.A. (2000). Conserved Structural Motifs Governing the Stoichiometric Repair of Alkylated DNA by O(6)-Alkylguanine-DNA Alkyltransferase. Mutat. Res..

[60] Zhang J., Stevens M.F.G., Bradshaw T.D. (2012). Temozolomide: Mechanisms of Action, Repair and Resistance. Curr. Mol. Pharmacol..

[61] Pataillot-Meakin T., Pillay N., Beck S. (2016). 3-Methylcytosine in Cancer: An Underappreciated Methyl Lesion?. Epigenomics.

[62] Furrer A., van Loon B. (2014). Handling the 3-Methylcytosine Lesion by Six Human DNA Polymerases Members of the B-, X- and Y-Families. Nucleic Acids Res..

[63] O’Brien P.J., Ellenberger T. (2004). Dissecting the Broad Substrate Specificity of Human 3-Methyladenine-DNA Glycosylase. J. Biol. Chem..

[64] Sedgwick B. (2004). Repairing DNA-Methylation Damage. Nat. Rev. Mol. Cell Biol..

[65] Dinglay S., Trewick S.C., Lindahl T., Sedgwick B. (2000). Defective Processing of Methylated Single-Stranded DNA by *E. coli* AlkB Mutants. Genes Dev..

[66] Trewick S.C., Henshaw T.F., Hausinger R.P., Lindahl T., Sedgwick B. (2002). Oxidative Demethylation by *Escherichia coli* AlkB Directly Reverts DNA Base Damage. Nature.

[67] Falnes P.Ø., Johansen R.F., Seeberg E. (2002). AlkB-Mediated Oxidative Demethylation Reverses DNA Damage in *Escherichia coli*. Nature.

[68] Kataoka H., Yamamoto Y., Sekiguchi M. (1983). A New Gene (alkB) of *Escherichia coli* That Controls Sensitivity to Methyl Methane Sulfonate. J. Bacteriol..

[69] Aravind L., Koonin E.V. (2001). The DNA-Repair Protein AlkB, EGL-9, and Leprecan Define New Families of 2-Oxoglutarate- and Iron-Dependent Dioxygenases. Genome Biol..

[70] Hausinger R.P. (2004). FeII/Alpha-Ketoglutarate-Dependent Hydroxylases and Related Enzymes. Crit. Rev. Biochem. Mol. Biol..

[71] Hernández-Caballero M.E., Sierra-Ramírez J.A. (2015). Single Nucleotide Polymorphisms of the FTO Gene and Cancer Risk: An Overview. Mol. Biol. Rep..

[72] Qu J., Yan H., Hou Y., Cao W., Liu Y., Zhang E., He J., Cai Z. (2022). RNA Demethylase ALKBH5 in Cancer: From Mechanisms to Therapeutic Potential. J. Hematol. Oncol..

[73] Wei H., Li Z., Liu F., Wang Y., Ding S., Chen Y., Liu J. (2022). The Role of FTO in Tumors and Its Research Progress. Curr. Med. Chem..

[74] Xu B., Liu D., Wang Z., Tian R., Zuo Y. (2021). Multi-Substrate Selectivity Based on Key Loops and Non-Homologous Domains: New Insight into ALKBH Family. Cell. Mol. Life Sci..

[75] Zhang Y., Wang C. (2021). Demethyltransferase AlkBH1 Substrate Diversity and Relationship to Human Diseases. Mol. Biol. Rep..

[76] Fu Y., Dominissini D., Rechavi G., He C. (2014). Gene Expression Regulation Mediated through Reversible m^6^A RNA Methylation. Nat. Rev. Genet..

[77] Fedeles B.I., Singh V., Delaney J.C., Li D., Essigmann J.M. (2015). The AlkB Family of Fe(II)/α-Ketoglutarate-Dependent Dioxygenases: Repairing Nucleic Acid Alkylation Damage and Beyond. J. Biol. Chem..

[78] Ougland R., Rognes T., Klungland A., Larsen E. (2015). Non-Homologous Functions of the AlkB Homologs. J. Mol. Cell Biol..

[79] Duncan T., Trewick S.C., Koivisto P., Bates P.A., Lindahl T., Sedgwick B. (2002). Reversal of DNA Alkylation Damage by Two Human Dioxygenases. Proc. Natl. Acad. Sci. USA.

[80] Gerken T., Girard C.A., Tung Y.-C.L., Webby C.J., Saudek V., Hewitson K.S., Yeo G.S.H., McDonough M.A., Cunliffe S., McNeill L.A. (2007). The Obesity-Associated FTO Gene Encodes a 2-Oxoglutarate-Dependent Nucleic Acid Demethylase. Science.

[81] Kurowski M.A., Bhagwat A.S., Papaj G., Bujnicki J.M. (2003). Phylogenomic Identification of Five New Human Homologs of the DNA Repair Enzyme AlkB. BMC Genom..

[82] Wei Y.F., Carter K.C., Wang R.P., Shell B.K. (1996). Molecular Cloning and Functional Analysis of a Human cDNA Encoding an *Escherichia coli* AlkB Homolog, a Protein Involved in DNA Alkylation Damage Repair. Nucleic Acids Res..

[83] Wang J., Wang J., Gu Q., Ma Y., Yang Y., Zhu J., Zhang Q. (2020). The Biological Function of m6A Demethylase ALKBH5 and Its Role in Human Disease. Cancer Cell Int..

[84] Fu D., Jordan J.J., Samson L.D. (2013). Human ALKBH7 Is Required for Alkylation and Oxidation-Induced Programmed Necrosis. Genes Dev..

[85] Songe-Møller L., van den Born E., Leihne V., Vågbø C.B., Kristoffersen T., Krokan H.E., Kirpekar F., Falnes P.Ø., Klungland A. (2010). Mammalian ALKBH8 Possesses tRNA Methyltransferase Activity Required for the Biogenesis of Multiple Wobble Uridine Modifications Implicated in Translational Decoding. Mol. Cell Biol..

[86] Khatiwada B., Nguyen T.T., Purslow J.A., Venditti V. (2022). Solution Structure Ensemble of Human Obesity-Associated Protein FTO Reveals Druggable Surface Pockets at the Interface between the N- and C-Terminal Domain. J. Biol. Chem..

[87] Han Z., Niu T., Chang J., Lei X., Zhao M., Wang Q., Cheng W., Wang J., Feng Y., Chai J. (2010). Crystal Structure of the FTO Protein Reveals Basis for Its Substrate Specificity. Nature.

[88] Alemu E.A., He C., Klungland A. (2016). ALKBHs-Facilitated RNA Modifications and de-Modifications. DNA Repair..

[89] Müller T.A., Meek K., Hausinger R.P. (2010). Human AlkB Homologue 1 (ABH1) Exhibits DNA Lyase Activity at Abasic Sites. DNA Repair..

[90] Korvald H., Falnes P.Ø., Laerdahl J.K., Bjørås M., Alseth I. (2012). The Schizosaccharomyces Pombe AlkB Homolog Abh1 Exhibits AP Lyase Activity but No Demethylase Activity. DNA Repair..

[91] Ma C.-J., Ding J.-H., Ye T.-T., Yuan B.-F., Feng Y.-Q. (2019). AlkB Homologue 1 Demethylates N3-Methylcytidine in mRNA of Mammals. ACS Chem. Biol..

[92] Wagner A., Hofmeister O., Rolland S.G., Maiser A., Aasumets K., Schmitt S., Schorpp K., Feuchtinger A., Hadian K., Schneider S. (2019). Mitochondrial Alkbh1 Localizes to mtRNA Granules and Its Knockdown Induces the Mitochondrial UPR in Humans and *C. elegans*. J. Cell Sci..

[93] Haag S., Sloan K.E., Ranjan N., Warda A.S., Kretschmer J., Blessing C., Hübner B., Seikowski J., Dennerlein S., Rehling P. (2016). NSUN 3 and ABH 1 Modify the Wobble Position of Mt-t RNA Met to Expand Codon Recognition in Mitochondrial Translation. EMBO J..

[94] Kawarada L., Suzuki T., Ohira T., Hirata S., Miyauchi K., Suzuki T. (2017). ALKBH1 Is an RNA Dioxygenase Responsible for Cytoplasmic and Mitochondrial tRNA Modifications. Nucleic Acids Res..

[95] Liu F., Clark W., Luo G., Wang X., Fu Y., Wei J., Wang X., Hao Z., Dai Q., Zheng G. (2016). ALKBH1-Mediated tRNA Demethylation Regulates Translation. Cell.

[96] Perry G.S., Das M., Woon E.C.Y. (2021). Inhibition of AlkB Nucleic Acid Demethylases: Promising New Epigenetic Targets. J. Med. Chem..

[97] Pan Z., Sikandar S., Witherspoon M., Dizon D., Nguyen T., Benirschke K., Wiley C., Vrana P., Lipkin S.M. (2008). Impaired Placental Trophoblast Lineage Differentiation in *Alkbh1*^−/−^ Mice. Dev. Dyn..

[98] Nordstrand L.M., Svärd J., Larsen E., Nilsen A., Ougland R., Furu K., Lien G.F., Rognes T., Namekawa S.H., Lee J.T. (2010). Mice Lacking Alkbh1 Display Sex-Ratio Distortion and Unilateral Eye Defects. PLoS ONE.

[99] Boos F., Wollin M., Herrmann J.M. (2016). Methionine on the Rise: How Mitochondria Changed Their Codon Usage. EMBO J..

[100] Li Q., Qian C., Feng H., Lin T., Zhu Q., Huang Y., Zhou F.-Q. (2021). N6-Methyladenine DNA Demethylase ALKBH1 Regulates Mammalian Axon Regeneration. Neurosci. Bull..

[101] Liu Y., Yuan Q., Xie L. (2018). The AlkB Family of Fe (II)/Alpha-Ketoglutarate-Dependent Dioxygenases Modulates Embryogenesis through Epigenetic Regulation. Curr. Stem Cell Res. Ther..

[102] Pilžys T., Marcinkowski M., Kukwa W., Garbicz D., Dylewska M., Ferenc K., Mieczkowski A., Kukwa A., Migacz E., Wołosz D. (2019). ALKBH Overexpression in Head and Neck Cancer: Potential Target for Novel Anticancer Therapy. Sci. Rep..

[103] Wang X., Wong C.C., Chen H., Fu K., Shi L., Su H., Guo S., Gou H., Hu X., Zhang L. (2023). The N6-Methyladenine DNA Demethylase ALKBH1 Promotes Gastric Carcinogenesis by Disrupting NRF1 Binding Capacity. Cell Rep..

[104] Chang R., Tsui K., Pan L., Li C. (2024). Spatial and Single-Cell Analyses Uncover Links between ALKBH1 and Tumor-Associated Macrophages in Gastric Cancer. Cancer Cell Int..

[105] Li Y., Zheng D., Wang F., Xu Y., Yu H., Zhang H. (2019). Expression of Demethylase Genes, FTO and ALKBH1, Is Associated with Prognosis of Gastric Cancer. Dig. Dis. Sci..

[106] Xie Q., Wu T.P., Gimple R.C., Li Z., Prager B.C., Wu Q., Yu Y., Wang P., Wang Y., Gorkin D.U. (2018). N6-Methyladenine DNA Modification in Glioblastoma. Cell.

[107] Zhang X., Zhou C., Zhao Y., Deng C., Wu H., Zhuo Z., He J. (2024). ALKBH1 Rs2267755 C>T Polymorphism Decreases Neuroblastoma Risk in Chinese Children. J. Cancer.

[108] Aas P.A., Otterlei M., Falnes P., Vågbø C.B., Skorpen F., Akbari M., Sundheim O., Bjørås M., Slupphaug G., Seeberg E. (2003). Human and Bacterial Oxidative Demethylases Repair Alkylation Damage in Both RNA and DNA. Nature.

[109] Falnes P., Bjørås M., Aas P.A., Sundheim O., Seeberg E. (2004). Substrate Specificities of Bacterial and Human AlkB Proteins. Nucleic Acids Res..

[110] Ringvoll J., Nordstrand L.M., Vagbo C.B., Talstad V., Reite K., Aas P.A., Lauritzen K.H., Liabakk N.B., Bjork A., Doughty R.W. (2006). Repair Deficient Mice Reveal mABH2 as the Primary Oxidative Demethylase for Repairing 1meA and 3meC Lesions in DNA. Embo J..

[111] Ringvoll J., Moen M.N., Nordstrand L.M., Meira L.B., Pang B., Bekkelund A., Dedon P.C., Bjelland S., Samson L.D., Falnes P.Ø. (2008). AlkB Homologue 2–Mediated Repair of Ethenoadenine Lesions in Mammalian DNA. Cancer Res..

[112] Zdzalik D., Domańska A., Prorok P., Kosicki K., van den Born E., Falnes P.T., Rizzo C.J., Guengerich F.P., Tudek B. (2015). Differential Repair of Etheno-DNA Adducts by Bacterial and Human AlkB Proteins. DNA Repair..

[113] Bian K., Lenz S.A.P., Tang Q., Chen F., Qi R., Jost M., Drennan C.L., Essigmann J.M., Wetmore S.D., Li D. (2019). DNA Repair Enzymes ALKBH2, ALKBH3, and AlkB Oxidize 5-Methylcytosine to 5-Hydroxymethylcytosine, 5-Formylcytosine and 5-Carboxylcytosine In Vitro. Nucleic Acids Res..

[114] Yi C., Yang C.-G., He C. (2009). A Non-Heme Iron-Mediated Chemical Demethylation in DNA and RNA. Acc. Chem. Res..

[115] Waheed S.O., Ramanan R., Chaturvedi S.S., Lehnert N., Schofield C.J., Christov C.Z., Karabencheva-Christova T.G. (2020). Role of Structural Dynamics in Selectivity and Mechanism of Non-Heme Fe(II) and 2-Oxoglutarate-Dependent Oxygenases Involved in DNA Repair. ACS Cent. Sci..

[116] You X.-J., Zhang S., Chen J.-J., Tang F., He J., Wang J., Qi C.-B., Feng Y.-Q., Yuan B.-F. (2022). Formation and Removal of 1,N6-Dimethyladenosine in Mammalian Transfer RNA. Nucleic Acids Res..

[117] Li P., Gao S., Wang L., Yu F., Li J., Wang C., Li J., Wong J. (2013). ABH2 Couples Regulation of Ribosomal DNA Transcription with DNA Alkylation Repair. Cell Rep..

[118] Wu Y., Chen Z., Xie G., Zhang H., Wang Z., Zhou J., Chen F., Li J., Chen L., Niu H. (2022). RNA m1A Methylation Regulates Glycolysis of Cancer Cells through Modulating ATP5D. Proc. Natl. Acad. Sci. USA.

[119] Chen Z., Qi M., Shen B., Luo G., Wu Y., Li J., Lu Z., Zheng Z., Dai Q., Wang H. (2019). Transfer RNA Demethylase ALKBH3 Promotes Cancer Progression via Induction of tRNA-Derived Small RNAs. Nucleic Acids Res..

[120] Cetica V., Genitori L., Giunti L., Sanzo M., Bernini G., Massimino M., Sardi I. (2009). Pediatric Brain Tumors: Mutations of Two Dioxygenases (hABH2 and hABH3) That Directly Repair Alkylation Damage. J. Neurooncol..

[121] Fu D., Samson L.D., Hübscher U., van Loon B. (2015). The Interaction between ALKBH2 DNA Repair Enzyme and PCNA Is Direct, Mediated by the Hydrophobic Pocket of PCNA and Perturbed in Naturally-Occurring ALKBH2 Variants. DNA Repair..

[122] Fujii T., Shimada K., Anai S., Fujimoto K., Konishi N. (2013). ALKBH2, a Novel AlkB Homologue, Contributes to Human Bladder Cancer Progression by Regulating MUC1 Expression. Cancer Sci..

[123] Ke B., Ye K., Cheng S. (2020). ALKBH2 Inhibition Alleviates Malignancy in Colorectal Cancer by Regulating BMI1-Mediated Activation of NF-κB Pathway. World J. Surg. Oncol..

[124] Wu S., Xu W., Liu S., Chen B., Wang X., Wang Y., Liu S., Wu J. (2011). Down-Regulation of ALKBH2 Increases Cisplatin Sensitivity in H1299 Lung Cancer Cells. Acta Pharmacol. Sin..

[125] Johannessen T.-C.A., Prestegarden L., Grudic A., Hegi M.E., Tysnes B.B., Bjerkvig R. (2013). The DNA Repair Protein ALKBH2 Mediates Temozolomide Resistance in Human Glioblastoma Cells. Neuro-Oncol..

[126] Konishi N., Shimada K., Nakamura M., Ishida E., Ota I., Tanaka N., Fujimoto K. (2008). Function of JunB in Transient Amplifying Cell Senescence and Progression of Human Prostate Cancer. Clin. Cancer Res..

[127] Tasaki M., Shimada K., Kimura H., Tsujikawa K., Konishi N. (2011). ALKBH3, a Human AlkB Homologue, Contributes to Cell Survival in Human Non-Small-Cell Lung Cancer. Br. J. Cancer.

[128] Nakao S., Mabuchi M., Shimizu T., Itoh Y., Takeuchi Y., Ueda M., Mizuno H., Shigi N., Ohshio I., Jinguji K. (2014). Design and Synthesis of Prostate Cancer Antigen-1 (PCA-1/ALKBH3) Inhibitors as Anti-Prostate Cancer Drugs. Bioorg. Med. Chem. Lett..

[129] Ueda M., Shimizu T., Mabuchi M., Horiike K., Kitae K., Hase H., Ueda Y., Tsujikawa K., Tanaka A. (2018). Novel Metabolically Stable PCA-1/ALKBH3 Inhibitor Has Potent Antiproliferative Effects on DU145 Cells In Vivo. Anticancer Res..

[130] Wang Q., Wang G., Wang Y., Liu C., He X. (2018). Association of AlkB Homolog 3 Expression with Tumor Recurrence and Unfavorable Prognosis in Hepatocellular Carcinoma. J. Gastroenterol. Hepatol..

[131] Shimada K., Fujii T., Tsujikawa K., Anai S., Fujimoto K., Konishi N. (2012). ALKBH3 Contributes to Survival and Angiogenesis of Human Urothelial Carcinoma Cells through NADPH Oxidase and Tweak/Fn14/VEGF Signals. Clin. Cancer Res..

[132] Bjørnstad L.G., Zoppellaro G., Tomter A.B., Falnes P., Andersson K.K. (2011). Spectroscopic and Magnetic Studies of Wild-Type and Mutant Forms of the Fe(II)- and 2-Oxoglutarate-Dependent Decarboxylase ALKBH4. Biochem. J..

[133] Li M.M., Nilsen A., Shi Y., Fusser M., Ding Y.H., Fu Y., Liu B., Niu Y., Wu Y.S., Huang C.M. (2013). ALKBH4-Dependent Demethylation of Actin Regulates Actomyosin Dynamics. Nat. Commun..

[134] Kweon S.-M., Chen Y., Moon E., Kvederaviciutė K., Klimasauskas S., Feldman D.E. (2019). An Adversarial DNA N6-Methyladenine-Sensor Network Preserves Polycomb Silencing. Mol. Cell.

[135] Nilsen A., Fusser M., Greggains G., Fedorcsak P., Klungland A. (2014). ALKBH4 Depletion in Mice Leads to Spermatogenic Defects. PLoS ONE.

[136] Sun Q., Liu X., Gong B., Wu D., Meng A., Jia S. (2017). Alkbh4 and Atrn Act Maternally to Regulate Zebrafish Epiboly. Int. J. Biol. Sci..

[137] Cui L., Fang L., Zhuang L., Shi B., Lin C.-P., Ye Y. (2023). Sperm-Borne microRNA-34c Regulates Maternal mRNA Degradation and Preimplantation Embryonic Development in Mice. Reprod. Biol. Endocrinol..

[138] Yu K., Qi T.F., Miao W., Liu X., Wang Y. (2022). Quantitative Proteomics Revealed New Functions of ALKBH4. Proteomics.

[139] Aoki M., Ueda K., Kamimura G., Iwamoto Y., Ikehata M., Tabata K., Sakagami Y., Morizono S., Tokunaga T., Umehara T. (2022). Clinical Significance of ALKBH4 Expression in Non-Small Cell Lung Cancer. Transl. Cancer Res..

[140] Peng B., Yan Y., Xu Z. (2021). The Bioinformatics and Experimental Analysis of AlkB Family for Prognosis and Immune Cell Infiltration in Hepatocellular Carcinoma. PeerJ.

[141] Jingushi K., Aoki M., Ueda K., Kogaki T., Tanimoto M., Monoe Y., Ando M., Matsumoto T., Minami K., Ueda Y. (2021). ALKBH4 Promotes Tumourigenesis with a Poor Prognosis in Non-Small-Cell Lung Cancer. Sci. Rep..

[142] Shen C., Yan T., Tong T., Shi D., Ren L., Zhang Y., Zhang X., Cao Y., Yan Y., Ma Y. (2020). ALKBH4 Functions as a Suppressor of Colorectal Cancer Metastasis via Competitively Binding to WDR5. Front. Cell Dev. Biol..

[143] Thalhammer A., Bencokova Z., Poole R., Loenarz C., Adam J., O’Flaherty L., Schödel J., Mole D., Giaslakiotis K., Schofield C.J. (2011). Human AlkB Homologue 5 Is a Nuclear 2-Oxoglutarate Dependent Oxygenase and a Direct Target of Hypoxia-Inducible Factor 1α (HIF-1α). PLoS ONE.

[144] Feng C., Liu Y., Wang G., Deng Z., Zhang Q., Wu W., Tong Y., Cheng C., Chen Z. (2014). Crystal Structures of the Human RNA Demethylase Alkbh5 Reveal Basis for Substrate Recognition. J. Biol. Chem..

[145] Shen F., Huang W., Huang J.-T., Xiong J., Yang Y., Wu K., Jia G.-F., Chen J., Feng Y.-Q., Yuan B.-F. (2015). Decreased N(6)-Methyladenosine in Peripheral Blood RNA from Diabetic Patients Is Associated with FTO Expression Rather than ALKBH5. J. Clin. Endocrinol. Metab..

[146] Xu C., Liu K., Tempel W., Demetriades M., Aik W., Schofield C.J., Min J. (2014). Structures of Human ALKBH5 Demethylase Reveal a Unique Binding Mode for Specific Single-Stranded N6-Methyladenosine RNA Demethylation. J. Biol. Chem..

[147] Zheng G., Dahl J.A., Niu Y., Fedorcsak P., Huang C.M., Li C.J., Vågbø C.B., Shi Y., Wang W.L., Song S.H. (2013). ALKBH5 Is a Mammalian RNA Demethylase That Impacts RNA Metabolism and Mouse Fertility. Mol. Cell.

[148] Akula D., O’Connor T.R., Anindya R. (2021). Oxidative Demethylase ALKBH5 Repairs DNA Alkylation Damage and Protects against Alkylation-Induced Toxicity. Biochem. Biophys. Res. Commun..

[149] Ensfelder T.T., Kurz M.Q., Iwan K., Geiger S., Matheisl S., Müller M., Beckmann R., Carell T. (2018). ALKBH5-Induced Demethylation of Mono- and Dimethylated Adenosine. Chem. Commun..

[150] Tang C., Klukovich R., Peng H., Wang Z., Yu T., Zhang Y., Zheng H., Klungland A., Yan W. (2018). ALKBH5-Dependent m6A Demethylation Controls Splicing and Stability of Long 3’-UTR mRNAs in Male Germ Cells. Proc. Natl. Acad. Sci. USA.

[151] Du T., Li G., Yang J., Ma K. (2020). RNA Demethylase Alkbh5 Is Widely Expressed in Neurons and Decreased during Brain Development. Brain Res. Bull..

[152] Meyer K.D., Jaffrey S.R. (2014). The Dynamic Epitranscriptome: N6-Methyladenosine and Gene Expression Control. Nat. Rev. Mol. Cell Biol..

[153] Yu F., Wei J., Cui X., Yu C., Ni W., Bungert J., Wu L., He C., Qian Z. (2021). Post-Translational Modification of RNA m6A Demethylase ALKBH5 Regulates ROS-Induced DNA Damage Response. Nucleic Acids Res..

[154] Zhou J., Zhang X., Hu J., Qu R., Yu Z., Xu H., Chen H., Yan L., Ding C., Zou Q. (2021). m6A Demethylase ALKBH5 Controls CD4+ T Cell Pathogenicity and Promotes Autoimmunity. Sci. Adv..

[155] Ren H., Zhuo Z.-J., Duan F., Li Y., Yang Z., Zhang J., Cheng J., Li S., Li L., Geng J. (2021). ALKBH5 Gene Polymorphisms and Hepatoblastoma Susceptibility in Chinese Children. J. Oncol..

[156] Guan Q., Lin H., Hua W., Lin L., Liu J., Deng L., Zhang J., Cheng J., Yang Z., Li Y. (2023). Variant Rs8400 Enhances ALKBH5 Expression through Disrupting miR-186 Binding and Promotes Neuroblastoma Progression. Chin. J. Cancer Res..

[157] Song R.-H., Zhao J., Gao C.-Q., Qin Q., Zhang J.-A. (2021). Inclusion of ALKBH5 as a Candidate Gene for the Susceptibility of Autoimmune Thyroid Disease. Adv. Med. Sci..

[158] Panneerdoss S., Eedunuri V.K., Yadav P., Timilsina S., Rajamanickam S., Viswanadhapalli S., Abdelfattah N., Onyeagucha B.C., Cui X., Lai Z. (2018). Cross-Talk among Writers, Readers, and Erasers of m6A Regulates Cancer Growth and Progression. Sci. Adv..

[159] Chao Y., Shang J., Ji W. (2020). ALKBH5-m6A-FOXM1 Signaling Axis Promotes Proliferation and Invasion of Lung Adenocarcinoma Cells under Intermittent Hypoxia. Biochem. Biophys. Res. Commun..

[160] Kowalski-Chauvel A., Lacore M.G., Arnauduc F., Delmas C., Toulas C., Cohen-Jonathan-Moyal E., Seva C. (2020). The m6A RNA Demethylase ALKBH5 Promotes Radioresistance and Invasion Capability of Glioma Stem Cells. Cancers.

[161] Zhang S., Zhao B.S., Zhou A., Lin K., Zheng S., Lu Z., Chen Y., Sulman E.P., Xie K., Bögler O. (2017). m6A Demethylase ALKBH5 Maintains Tumorigenicity of Glioblastoma Stem-like Cells by Sustaining FOXM1 Expression and Cell Proliferation Program. Cancer Cell.

[162] Nagaki Y., Motoyama S., Yamaguchi T., Hoshizaki M., Sato Y., Sato T., Koizumi Y., Wakita A., Kawakita Y., Imai K. (2020). M6 A Demethylase ALKBH5 Promotes Proliferation of Esophageal Squamous Cell Carcinoma Associated with Poor Prognosis. Genes Cells.

[163] Zhang C., Samanta D., Lu H., Bullen J.W., Zhang H., Chen I., He X., Semenza G.L. (2016). Hypoxia Induces the Breast Cancer Stem Cell Phenotype by HIF-Dependent and ALKBH5-Mediated m^6^A-Demethylation of NANOG mRNA. Proc. Natl. Acad. Sci. USA.

[164] Han X., Ren C., Jiang A., Sun Y., Lu J., Ling X., Lu C., Yu Z. (2024). Arginine Methylation of ALKBH5 by PRMT6 Promotes Breast Tumorigenesis via LDHA-Mediated Glycolysis. Front. Med..

[165] Zhao Y., Hu J., Sun X., Yang K., Yang L., Kong L., Zhang B., Li F., Li C., Shi B. (2021). Loss of m6A Demethylase ALKBH5 Promotes Post-Ischemic Angiogenesis via Post-Transcriptional Stabilization of WNT5A. Clin. Transl. Med..

[166] Li X.-C., Jin F., Wang B.-Y., Yin X.-J., Hong W., Tian F.-J. (2019). The m6A Demethylase ALKBH5 Controls Trophoblast Invasion at the Maternal-Fetal Interface by Regulating the Stability of CYR61 mRNA. Theranostics.

[167] Chen Y., Zhao Y., Chen J., Peng C., Zhang Y., Tong R., Cheng Q., Yang B., Feng X., Lu Y. (2020). ALKBH5 Suppresses Malignancy of Hepatocellular Carcinoma via m6A-Guided Epigenetic Inhibition of LYPD1. Mol. Cancer.

[168] Yu H., Yang X., Tang J., Si S., Zhou Z., Lu J., Han J., Yuan B., Wu Q., Lu Q. (2021). ALKBH5 Inhibited Cell Proliferation and Sensitized Bladder Cancer Cells to Cisplatin by m6A-CK2α-Mediated Glycolysis. Mol. Ther. Nucleic Acids.

[169] Yuan Y., Yan G., He M., Lei H., Li L., Wang Y., He X., Li G., Wang Q., Gao Y. (2021). ALKBH5 Suppresses Tumor Progression via an m6A-Dependent Epigenetic Silencing of Pre-miR-181b-1/YAP Signaling Axis in Osteosarcoma. Cell Death Dis..

[170] Luo Q., Gao Y., Zhang L., Rao J., Guo Y., Huang Z., Li J. (2020). Decreased ALKBH5, FTO, and YTHDF2 in Peripheral Blood Are as Risk Factors for Rheumatoid Arthritis. BioMed Res. Int..

[171] Selberg S., Seli N., Kankuri E., Karelson M. (2021). Rational Design of Novel Anticancer Small-Molecule RNA m6A Demethylase ALKBH5 Inhibitors. ACS Omega.

[172] You Y., Fu Y., Huang M., Shen D., Zhao B., Liu H., Zheng Y., Huang L. (2022). Recent Advances of m6A Demethylases Inhibitors and Their Biological Functions in Human Diseases. Int. J. Mol. Sci..

[173] Tsujikawa K., Koike K., Kitae K., Shinkawa A., Arima H., Suzuki T., Tsuchiya M., Makino Y., Furukawa T., Konishi N. (2007). Expression and Sub-Cellular Localization of Human ABH Family Molecules. J. Cell Mol. Med..

[174] Huong T.T., Ngoc L.N.T., Kang H. (2020). Functional Characterization of a Putative RNA Demethylase Alkbh6 in Arabidopsis Growth and Abiotic Stress Responses. Int. J. Mol. Sci..

[175] Ma L., Lu H., Tian Z., Yang M., Ma J., Shang G., Liu Y., Xie M., Wang G., Wu W. (2022). Structural Insights into the Interactions and Epigenetic Functions of Human Nucleic Acid Repair Protein ALKBH6. J. Biol. Chem..

[176] Zhao S., Devega R., Francois A., Kidane D. (2021). Human ALKBH6 Is Required for Maintenance of Genomic Stability and Promoting Cell Survival during Exposure of Alkylating Agents in Pancreatic Cancer. Front. Genet..

[177] Solberg A., Robertson A.B., Aronsen J.M., Rognmo Ø., Sjaastad I., Wisløff U., Klungland A. (2013). Deletion of Mouse Alkbh7 Leads to Obesity. J. Mol. Cell Biol..

[178] Wang G., He Q., Feng C., Liu Y., Deng Z., Qi X., Wu W., Mei P., Chen Z. (2014). The Atomic Resolution Structure of Human AlkB Homolog 7 (ALKBH7), a Key Protein for Programmed Necrosis and Fat Metabolism. J. Biol. Chem..

[179] Zhang L.-S., Xiong Q.-P., Peña Perez S., Liu C., Wei J., Le C., Zhang L., Harada B.T., Dai Q., Feng X. (2021). ALKBH7-Mediated Demethylation Regulates Mitochondrial Polycistronic RNA Processing. Nat. Cell Biol..

[180] Walker A.R., Silvestrov P., Müller T.A., Podolsky R.H., Dyson G., Hausinger R.P., Cisneros G.A. (2017). ALKBH7 Variant Related to Prostate Cancer Exhibits Altered Substrate Binding. PLoS Comput. Biol..

[181] Begley U., Dyavaiah M., Patil A., Rooney J.P., DiRenzo D., Young C.M., Conklin D.S., Zitomer R.S., Begley T.J. (2007). Trm9-Catalyzed tRNA Modifications Link Translation to the DNA Damage Response. Mol. Cell.

[182] Shimada K., Nakamura M., Anai S., De Velasco M., Tanaka M., Tsujikawa K., Ouji Y., Konishi N. (2009). A Novel Human AlkB Homologue, ALKBH8, Contributes to Human Bladder Cancer Progression. Cancer Res..

[183] Fu D., Brophy J.A.N., Chan C.T.Y., Atmore K.A., Begley U., Paules R.S., Dedon P.C., Begley T.J., Samson L.D. (2010). Human AlkB Homolog ABH8 Is a tRNA Methyltransferase Required for Wobble Uridine Modification and DNA Damage Survival. Mol. Cell Biol..

[184] Pastore C., Topalidou I., Forouhar F., Yan A.C., Levy M., Hunt J.F. (2012). Crystal Structure and RNA Binding Properties of the RNA Recognition Motif (RRM) and AlkB Domains in Human AlkB Homolog 8 (ABH8), an Enzyme Catalyzing tRNA Hypermodification. J. Biol. Chem..

[185] Fu Y., Dai Q., Zhang W., Ren J., Pan T., He C. (2010). The AlkB Domain of Mammalian ABH8 Catalyzes Hydroxylation of 5-Methoxycarbonylmethyluridine at the Wobble Position of tRNA. Angew. Chem. Int. Ed. Engl..

[186] Van Den Born E., Vågbø C.B., Songe-Møller L., Leihne V., Lien G.F., Leszczynska G., Malkiewicz A., Krokan H.E., Kirpekar F., Klungland A. (2011). ALKBH8-Mediated Formation of a Novel Diastereomeric Pair of Wobble Nucleosides in Mammalian tRNA. Nat. Commun..

[187] Madhwani K.R., Sayied S., Ogata C.H., Hogan C.A., Lentini J.M., Mallik M., Dumouchel J.L., Storkebaum E., Fu D., O’Connor-Giles K.M. (2023). tRNA Modification Enzyme-Dependent Redox Homeostasis Regulates Synapse Formation and Memory. bioRxiv.

[188] Monies D., Vågbø C.B., Al-Owain M., Alhomaidi S., Alkuraya F.S. (2019). Recessive Truncating Mutations in ALKBH8 Cause Intellectual Disability and Severe Impairment of Wobble Uridine Modification. Am. J. Hum. Genet..

[189] Saad A.K., Marafi D., Mitani T., Du H., Rafat K., Fatih J.M., Jhangiani S.N., Coban-Akdemir Z., Gibbs R.A., Baylor-Hopkins Center for Mendelian Genomics (2021). Neurodevelopmental Disorder in an Egyptian Family with a Biallelic ALKBH8 Variant. Am. J. Med. Genet. Part A.

[190] Maddirevula S., Alameer S., Ewida N., de Sousa M.M.L., Bjørås M., Vågbø C.B., Alkuraya F.S. (2022). Insight into ALKBH8-Related Intellectual Developmental Disability Based on the First Pathogenic Missense Variant. Hum. Genet..

[191] Yılmaz M., Kamaşak T., Teralı K., Çebi A.H., Türkyılmaz A. (2024). The First Turkish Family with a Novel Biallelic Missense Variant of the ALKBH8 Gene: A Study on the Clinical and Variant Spectrum of ALKBH8-Related Intellectual Developmental Disorders. Am. J. Med. Genet. Part A.

[192] Waqas A., Nayab A., Shaheen S., Abbas S., Latif M., Rafeeq M.M., Al-Dhuayan I.S., Alqosaibi A.I., Alnamshan M.M., Sain Z.M. (2022). Case Report: Biallelic Variant in the tRNA Methyltransferase Domain of the AlkB Homolog 8 Causes Syndromic Intellectual Disability. Front. Genet..

[193] Ohshio I., Kawakami R., Tsukada Y., Nakajima K., Kitae K., Shimanoe T., Saigo Y., Hase H., Ueda Y., Jingushi K. (2016). ALKBH8 Promotes Bladder Cancer Growth and Progression through Regulating the Expression of Survivin. Biochem. Biophys. Res. Commun..

[194] Ueda Y., Ooshio I., Fusamae Y., Kitae K., Kawaguchi M., Jingushi K., Hase H., Harada K., Hirata K., Tsujikawa K. (2017). AlkB Homolog 3-Mediated tRNA Demethylation Promotes Protein Synthesis in Cancer Cells. Sci. Rep..

[195] Jia G., Yang C.-G., Yang S., Jian X., Yi C., Zhou Z., He C. (2008). Oxidative Demethylation of 3-Methylthymine and 3-Methyluracil in Single-Stranded DNA and RNA by Mouse and Human FTO. FEBS Lett..

[196] Jia G., Fu Y., Zhao X., Dai Q., Zheng G., Yang Y., Yi C., Lindahl T., Pan T., Yang Y.-G. (2011). N6-Methyladenosine in Nuclear RNA Is a Major Substrate of the Obesity-Associated FTO. Nat. Chem. Biol..

[197] Desrosiers R., Friderici K., Rottman F. (1974). Identification of Methylated Nucleosides in Messenger RNA from Novikoff Hepatoma Cells. Proc. Natl. Acad. Sci. USA.

[198] Kowalak J.A., Pomerantz S.C., Crain P.F., McCloskey J.A. (1993). A Novel Method for the Determination of Post-Transcriptional Modification in RNA by Mass Spectrometry. Nucleic Acids Res..

[199] Yeo G.S.H. (2014). The Role of the FTO (Fat Mass and Obesity Related) Locus in Regulating Body Size and Composition. Mol. Cell Endocrinol..

[200] Li Y., Wu K., Quan W., Yu L., Chen S., Cheng C., Wu Q., Zhao S., Zhang Y., Zhou L. (2019). The Dynamics of FTO Binding and Demethylation from the m6A Motifs. RNA Biol..

[201] Meyre D., Proulx K., Kawagoe-Takaki H., Vatin V., Gutiérrez-Aguilar R., Lyon D., Ma M., Choquet H., Horber F., Van Hul W. (2010). Prevalence of Loss-of-Function FTO Mutations in Lean and Obese Individuals. Diabetes.

[202] Yeo G.S.H., Heisler L.K. (2012). Unraveling the Brain Regulation of Appetite: Lessons from Genetics. Nat. Neurosci..

[203] Walters B.J., Mercaldo V., Gillon C.J., Yip M., Neve R.L., Boyce F.M., Frankland P.W., Josselyn S.A. (2017). The Role of The RNA Demethylase FTO (Fat Mass and Obesity-Associated) and mRNA Methylation in Hippocampal Memory Formation. Neuropsychopharmacology.

[204] Cao Y., Zhuang Y., Chen J., Xu W., Shou Y., Huang X., Shu Q., Li X. (2020). Dynamic Effects of Fto in Regulating the Proliferation and Differentiation of Adult Neural Stem Cells of Mice. Hum. Mol. Genet..

[205] Sun X., Zhang Y., Hu Y., An J., Li L., Wang Y., Zhang X. (2021). Decreased Expression of m6A Demethylase FTO in Ovarian Aging. Arch. Gynecol. Obs..

[206] Liu S., Xiu J., Zhu C., Meng K., Li C., Han R., Du T., Li L., Xu L., Liu R. (2021). Fat Mass and Obesity-Associated Protein Regulates RNA Methylation Associated with Depression-like Behavior in Mice. Nat. Commun..

[207] Wu R., Chen Y., Liu Y., Zhuang L., Chen W., Zeng B., Liao X., Guo G., Wang Y., Wang X. (2021). m6A Methylation Promotes White-to-Beige Fat Transition by Facilitating Hif1a Translation. EMBO Rep..

[208] Zhang M., Zhang Y., Ma J., Guo F., Cao Q., Zhang Y., Zhou B., Chai J., Zhao W., Zhao R. (2015). The Demethylase Activity of FTO (Fat Mass and Obesity Associated Protein) Is Required for Preadipocyte Differentiation. PLoS ONE.

[209] Sun D., Zhao T., Zhang Q., Wu M., Zhang Z. (2021). Fat Mass and Obesity-Associated Protein Regulates Lipogenesis via M6 A Modification in Fatty Acid Synthase mRNA. Cell Biol. Int..

[210] Wang X., Huang N., Yang M., Wei D., Tai H., Han X., Gong H., Zhou J., Qin J., Wei X. (2017). FTO Is Required for Myogenesis by Positively Regulating mTOR-PGC-1α Pathway-Mediated Mitochondria Biogenesis. Cell Death Dis..

[211] Frayling T.M., Timpson N.J., Weedon M.N., Zeggini E., Freathy R.M., Lindgren C.M., Perry J.R.B., Elliott K.S., Lango H., Rayner N.W. (2007). A Common Variant in the FTO Gene Is Associated with Body Mass Index and Predisposes to Childhood and Adult Obesity. Science.

[212] Olmedo L., Luna F.J., Zubrzycki J., Dopazo H., Pellon-Maison M. (2024). Associations between Rs9939609 FTO Polymorphism with Nutrient and Food Intake and Adherence to Dietary Patterns in an Urban Argentinian Population. J. Acad. Nutr. Diet..

[213] Church C., Moir L., McMurray F., Girard C., Banks G.T., Teboul L., Wells S., Brüning J.C., Nolan P.M., Ashcroft F.M. (2010). Overexpression of Fto Leads to Increased Food Intake and Results in Obesity. Nat. Genet..

[214] Goutzelas Y., Kotsa K., Vasilopoulos Y., Tsekmekidou X., Stamatis C., Yovos J.G., Sarafidou T., Mamuris Z. (2017). Association Analysis of FTO Gene Polymorphisms with Obesity in Greek Adults. Gene.

[215] Church C., Lee S., Bagg E.A.L., McTaggart J.S., Deacon R., Gerken T., Lee A., Moir L., Mecinović J., Quwailid M.M. (2009). A Mouse Model for the Metabolic Effects of the Human Fat Mass and Obesity Associated FTO Gene. PLoS Genet..

[216] Fischer J., Koch L., Emmerling C., Vierkotten J., Peters T., Brüning J.C., Rüther U. (2009). Inactivation of the Fto Gene Protects from Obesity. Nature.

[217] McMurray F., Church C.D., Larder R., Nicholson G., Wells S., Teboul L., Tung Y.C.L., Rimmington D., Bosch F., Jimenez V. (2013). Adult Onset Global Loss of the Fto Gene Alters Body Composition and Metabolism in the Mouse. PLoS Genet..

[218] Boissel S., Reish O., Proulx K., Kawagoe-Takaki H., Sedgwick B., Yeo G.S.H., Meyre D., Golzio C., Molinari F., Kadhom N. (2009). Loss-of-Function Mutation in the Dioxygenase-Encoding FTO Gene Causes Severe Growth Retardation and Multiple Malformations. Am. J. Hum. Genet..

[219] Daoud H., Zhang D., McMurray F., Yu A., Luco S.M., Vanstone J., Jarinova O., Carson N., Wickens J., Shishodia S. (2016). Identification of a Pathogenic FTO Mutation by Next-Generation Sequencing in a Newborn with Growth Retardation and Developmental Delay. J. Med. Genet..

[220] Rohena L., Lawson M., Guzman E., Ganapathi M., Cho M.T., Haverfield E., Anyane-Yeboa K. (2016). FTO Variant Associated with Malformation Syndrome. Am. J. Med. Genet. Part A.

[221] Landfors M., Nakken S., Fusser M., Dahl J.-A., Klungland A., Fedorcsak P. (2016). Sequencing of FTO and ALKBH5 in Men Undergoing Infertility Work-up Identifies an Infertility-Associated Variant and Two Missense Mutations. Fertil. Steril..

[222] Mayman N., Wei J., Cai S., Soman R., Raynes H., La Vega-Talbott M., He C., Naidich T., Raju G.P., Kathiresu Nageshwaran S. (2023). Case Report: A Novel Biallelic FTO Variant Causing Multisystem Anomalies with Severe Epilepsy, Widening the Spectrum of FTO Syndrome. SAGE Open Med. Case Rep..

[223] Zou D., Dong L., Li C., Yin Z., Rao S., Zhou Q. (2019). The m6A Eraser FTO Facilitates Proliferation and Migration of Human Cervical Cancer Cells. Cancer Cell Int..

[224] Niu Y., Lin Z., Wan A., Chen H., Liang H., Sun L., Wang Y., Li X., Xiong X.-F., Wei B. (2019). RNA N6-Methyladenosine Demethylase FTO Promotes Breast Tumor Progression through Inhibiting BNIP3. Mol. Cancer.

[225] Yang S., Wei J., Cui Y.-H., Park G., Shah P., Deng Y., Aplin A.E., Lu Z., Hwang S., He C. (2019). m6A mRNA Demethylase FTO Regulates Melanoma Tumorigenicity and Response to Anti-PD-1 Blockade. Nat. Commun..

[226] Liu Y., Liang G., Xu H., Dong W., Dong Z., Qiu Z., Zhang Z., Li F., Huang Y., Li Y. (2021). Tumors Exploit FTO-Mediated Regulation of Glycolytic Metabolism to Evade Immune Surveillance. Cell Metab..

[227] Tian R., Zhang S., Sun D., Bei C., Li D., Zheng C., Song X., Chen M., Tan S., Zhu X. (2020). M6A Demethylase FTO Plays a Tumor Suppressor Role in Thyroid Cancer. DNA Cell Biol..

[228] Claussnitzer M., Dankel S.N., Kim K.-H., Quon G., Meuleman W., Haugen C., Glunk V., Sousa I.S., Beaudry J.L., Puviindran V. (2015). FTO Obesity Variant Circuitry and Adipocyte Browning in Humans. N. Engl. J. Med..

[229] Dina C., Meyre D., Gallina S., Durand E., Körner A., Jacobson P., Carlsson L.M.S., Kiess W., Vatin V., Lecoeur C. (2007). Variation in FTO Contributes to Childhood Obesity and Severe Adult Obesity. Nat. Genet..

[230] Smemo S., Tena J.J., Kim K.-H., Gamazon E.R., Sakabe N.J., Gómez-Marín C., Aneas I., Credidio F.L., Sobreira D.R., Wasserman N.F. (2014). Obesity-Associated Variants within FTO Form Long-Range Functional Connections with IRX3. Nature.

[231] Freathy R.M., Timpson N.J., Lawlor D.A., Pouta A., Ben-Shlomo Y., Ruokonen A., Ebrahim S., Shields B., Zeggini E., Weedon M.N. (2008). Common Variation in the FTO Gene Alters Diabetes-Related Metabolic Traits to the Extent Expected given Its Effect on BMI. Diabetes.

[232] Scuteri A., Sanna S., Chen W.-M., Uda M., Albai G., Strait J., Najjar S., Nagaraja R., Orrú M., Usala G. (2007). Genome-Wide Association Scan Shows Genetic Variants in the FTO Gene Are Associated with Obesity-Related Traits. PLoS Genet..

[233] Keller L., Xu W., Wang H.-X., Winblad B., Fratiglioni L., Graff C. (2011). The Obesity Related Gene, FTO, Interacts with APOE, and Is Associated with Alzheimer’s Disease Risk: A Prospective Cohort Study. J. Alzheimers. Dis..

[234] Reitz C., Tosto G., Mayeux R., Luchsinger J.A., NIA-LOAD/NCRAD Family Study Group, Alzheimer’s Disease Neuroimaging Initiative (2012). Genetic Variants in the Fat and Obesity Associated (FTO) Gene and Risk of Alzheimer’s Disease. PLoS ONE.

[235] Lim A., Zhou J., Sinha R.A., Singh B.K., Ghosh S., Lim K.-H., Chow P.K.-H., Woon E.C.Y., Yen P.M. (2016). Hepatic FTO Expression Is Increased in NASH and Its Silencing Attenuates Palmitic Acid-Induced Lipotoxicity. Biochem. Biophys. Res. Commun..

[236] Huang Y., Su R., Sheng Y., Dong L., Dong Z., Xu H., Ni T., Zhang Z.S., Zhang T., Li C. (2019). Small-Molecule Targeting of Oncogenic FTO Demethylase in Acute Myeloid Leukemia. Cancer Cell.

[237] Zou S., Toh J.D.W., Wong K.H.Q., Gao Y.-G., Hong W., Woon E.C.Y. (2016). N(6)-Methyladenosine: A Conformational Marker That Regulates the Substrate Specificity of Human Demethylases FTO and ALKBH5. Sci. Rep..

